# Discovery of Human Inversion Polymorphisms by Comparative Analysis of Human and Chimpanzee DNA Sequence Assemblies

**DOI:** 10.1371/journal.pgen.0010056

**Published:** 2005-10-28

**Authors:** Lars Feuk, Jeffrey R MacDonald, Terence Tang, Andrew R Carson, Martin Li, Girish Rao, Razi Khaja, Stephen W Scherer

**Affiliations:** 1 The Centre for Applied Genomics, Department of Genetics and Genomic Biology, The Hospital for Sick Children, Toronto, Ontario, Canada; 2 Department of Molecular and Medical Genetics, University of Toronto, Ontario, Canada; Fred Hutchinson Cancer Research Center, United States of America

## Abstract

With a draft genome-sequence assembly for the chimpanzee available, it is now possible to perform genome-wide analyses to identify, at a submicroscopic level, structural rearrangements that have occurred between chimpanzees and humans. The goal of this study was to investigate chromosomal regions that are inverted between the chimpanzee and human genomes. Using the net alignments for the builds of the human and chimpanzee genome assemblies, we identified a total of 1,576 putative regions of inverted orientation, covering more than 154 mega-bases of DNA. The DNA segments are distributed throughout the genome and range from 23 base pairs to 62 mega-bases in length. For the 66 inversions more than 25 kilobases (kb) in length, 75% were flanked on one or both sides by (often unrelated) segmental duplications. Using PCR and fluorescence in situ hybridization we experimentally validated 23 of 27 (85%) semi-randomly chosen regions; the largest novel inversion confirmed was 4.3 mega-bases at human Chromosome 7p14. Gorilla was used as an out-group to assign ancestral status to the variants. All experimentally validated inversion regions were then assayed against a panel of human samples and three of the 23 (13%) regions were found to be polymorphic in the human genome. These polymorphic inversions include 730 kb (at 7p22), 13 kb (at 7q11), and 1 kb (at 16q24) fragments with a 5%, 30%, and 48% minor allele frequency, respectively. Our results suggest that inversions are an important source of variation in primate genome evolution. The finding of at least three novel inversion polymorphisms in humans indicates this type of structural variation may be a more common feature of our genome than previously realized.

## Synopsis

Chimpanzee is the closest relative to humans having DNA sequences about 98% identical to each other. Small DNA sequence changes and probably more importantly, larger structural changes of chromosomes, led to the divergence of the two species some 6 million years ago. Until recently, there were ten structural differences visible under the microscope between chimpanzee and human, and nine of these were inversions of DNA. Through computational comparisons of genome sequences, the current study identifies another 1,576 putative inversion events. Thirty-three of these were larger than 100,000 base pairs in size and 29 intersect genes, prioritizing them for evolutionary studies. Twenty-three of the inversions have been confirmed experimentally with the largest being 4.3 million base pairs in size on human Chromosome 7. Surprisingly, three of the “inverted” regions were found to be variable in their orientation in the human population (in some cases the inversion was in the ancestral orientation found in chimpanzee). These observations indicate the human genome is still evolving in structure. Moreover, since such variable inversions have been shown to predispose to other (sometimes deleterious) changes in chromosomes, the new data delineate potential disease-associated genes.

## Introduction

Humans and chimpanzees diverged approximately 6 million years ago, making the chimpanzee the closest extant relative to modern humans. The characterization of sequence changes both at the nucleotide and the structural level is therefore important for the understanding of primate evolution, including human-specific traits. At the nucleotide level, the identity of the genomes has been estimated to be 98% to 99% [1–5], excluding insertions and deletions and other small rearrangements. The chimpanzee Chromosome 22 (PTR22), which is orthologous to human Chromosome 21(HSA21), was the first to be sequenced and the majority of the rest of the genome is represented as a draft assembly [2,6]. The exact nucleotide substitution rate for the alignment of these sequences is 1.23% (excluding insertions and deletions)[2]. Taking insertion and deletion events into account, the sequence identity has been estimated to be about 95% [7].

In addition to nucleotide level changes, large structural rearrangements have also occurred between the species and they are discernable through comparison of the G-banded karyotypes. The most obvious structural difference between the human and chimpanzee genomes is the fusion of two acrocentric chromosomes creating human Chromosome 2. This results in a lower total chromosome number in humans (22, XY versus 23, XY). In addition, there are nine visible pericentric inversions affecting Chromosomes 1, 4, 5, 9, 12, 15, 16, 17, and 18 [8]. Of these rearrangements, only the fusion creating Chromosome 2 and the inversions on Chromosome 1 and 18 are specific to the human lineage, while the remaining changes have occurred in the chimpanzee lineage.

Early comparative studies between the human and chimpanzee genomes focused mainly on localized sequencing efforts and characterization of karyotypically visible chromosomal rearrangements. More recently, a number of studies have been performed with the goal of characterizing loss and gain of submicroscopic regions of DNA using comparative genomic hybridization [9,10]. The results reveal that copy number differences are abundant between the human and chimpanzee genomes, which agree with studies of segmental duplications in the genomes of several species [11–13]. These latter studies show that there is a higher incidence of segmental duplications in the human genome than in the mouse or rat genomes, indicating that increases in sequence copy number are more common in recent primate evolution [14]. The high frequency of copy number differences between humans and chimpanzees are also consistent with the findings that these types of structural variants are present as a common type of polymorphism in the human genome [15–19].

Although recent technological advances allow for detection of most types of genomic variation, limitations in available methodology have prevented the genome-wide discovery of balanced rearrangements such as inversions. Nonetheless, the fact that nine known cytogenetically visible inversion events distinguish the human and chimpanzee genomes indicates that these may have been a common form of structural rearrangement during primate evolution. The comparative study of human Chromosome 21 and chimpanzee Chromosome 22 did not assess the extent of inversion events between the two species [6]. The recent publication of the chimpanzee genome and accompanying comparative analysis of structural rearrangements did not address inversion events beyond those that are visible in the karyotype [2,16].

Characterization of inversion events between humans and chimpanzees are important because inversions can affect the expression of genes adjacent to the breakpoints, or directly interrupt genes spanning the breakpoints. Large inversions have also been proposed to be a direct driving force in speciation [20] and have been shown to suppress recombination [21]. It is also important to investigate inversion events between humans and chimpanzees as the frequency of such events can provide an indication as to what extent inversion variants exist as polymorphisms in the human population. Since inversion polymorphisms are difficult to detect, there has not been, until very recently, an estimate of their occurrence in the human genome. By mapping fosmid ends to the reference genome sequence, Tuzun et al. identified 56 putative inversion breakpoints in a single individual (inversion breakpoint pairs cannot be identified unambiguously using this approach). This implies that inversion polymorphisms are much more common than previously assumed.

Using the draft sequence of *Pan troglodytes* (chimpanzee), we have used alignments between the human and chimpanzee genomes to identify regions of inverted orientation. Through this computational approach, there is no theoretical limit to the size resolution of inversion regions that can be identified and resolved. It is, therefore, possible to equally identify cytogenetically detectable, as well as nucleotide level inversions, with a resolution down to the breakpoint sequence itself. Of the 1,576 computationally predicted inversions, 23 have now been confirmed experimentally. Screening in human control individuals also revealed three regions to be polymorphic in the human population. Our data indicate that inversions have occurred frequently in recent primate evolution, and both computational analysis and experimental data support the observation that inversion polymorphisms may be common in the human genome.

## Results

### Computational Analysis for Identification of Putative Inversions

**Figure 1:**
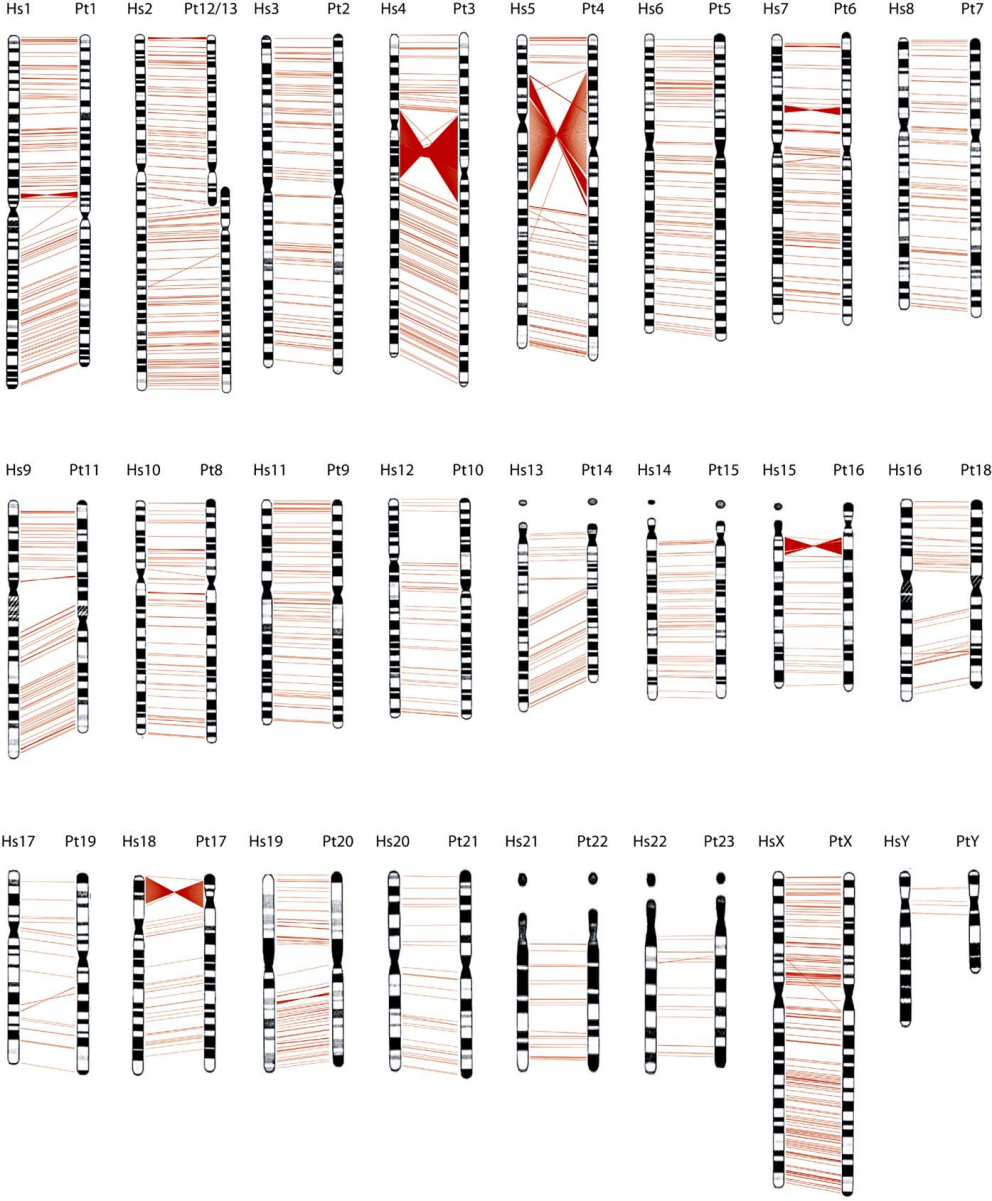
Genome-Wide Distribution of the 1,576 Putative Inversions Identified between the Human and Chimpanzee Assemblies Human chromosomes are shown to the left and the syntenic chimpanzee chromosome to the right. Each red line corresponds to an inversion between the human and chimpanzee assemblies. Regions larger than 100 kb are represented with multiple lines. These include the large inversions on human Chromosome 4, 5, 15, and 18, while those on human Chromosomes 9, 12, and 17 were not identified. The karyotypically visible pericentric inversions on Chromosome 1 and 16 have not been described at the molecular level.

Net alignments of the human genome (assembly Build 35, hg_17) with the chimpanzee draft genome (assembly Build 1) were downloaded from the University of California, Santa Cruz (http://genome.ucsc.edu/). All alignments of inverted orientation more than 20 base pair (bp) in length were identified. Segmental duplication and repeats were identified as a source of non-syntenic alignments and all alignments with a repeat or duplication content of more than 90%, as well as regions where the net alignment and the reciprocal best-hits between human and chimpanzee sequences disagreed, were excluded. After filtering, 1,576 putative inversions were identified between the two genomes (Figure 1 and Table S1). In total, these regions cover more than 154 mega-bases (Mb) of DNA. In addition to the National Center for Biotechnology (NCBI) assembly Build 35 of the human genome, there is an independent assembly of human Chromosome 7 (CRA_TCAGchr7v2 from http://www.chr7.org) [22]. Using this assembly of Chromosome 7, another two regions of inverted orientation compared to the chimpanzee Chromosome 6 were identified.

**Figure 2:**
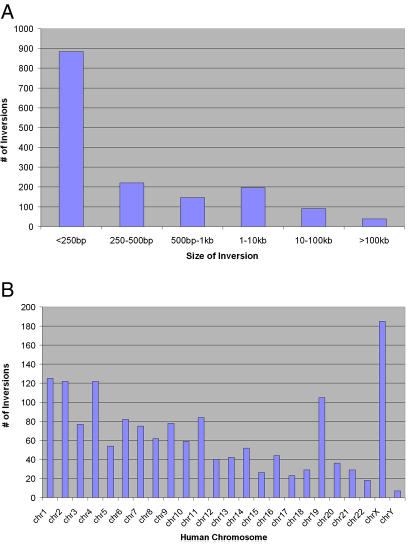
Size Distribution and Chromosomal Distribution of Putative Inversions (A) Size distribution of inversions. The size of the inversion regions identified range from 23 bp to 62 Mb, but more than half of all regions identified are less then 250 bp. The algorithm used to create the net alignments is more prone to make errors and assign random orientation to very short regions (Table S1 and Figure S1). However, we did not see this trend in the regions chosen for experimental validation. Thirty-three of the regions identified were larger than 100 kb in size. (B) Chromosomal distribution of inversion regions. The autosomal chromosomes have a distribution of inversions roughly correlated to the size of the chromosome, except for Chromosome 19 which carries approximately the same number as Chromosomes 1 to 4. The X chromosome also shows an increase of inversions compared to autosomes of corresponding size.

The inverted regions identified are distributed amongst and throughout the human chromosomes (Figure 2A), with the highest content being on the X chromosome. Inversions range in size from 23 bp to 62 Mb, with the largest regions representing the karyotypically visible pericentric inversions (Figure 2B). In total, 33 inversions larger than 100 kb were identified. Seven of the nine known pericentric inversions have been characterized at the molecular level, and four of these seven regions were identified in our analysis (on human Chromosomes 4, 5, 15, and 18; Tables S3 and S4). The previously characterized inversions on Chromosomes 9, 10, and 19 [8] are not present in the correct orientation in the current draft of the chimpanzee assembly, presumably since these chromosomes still contain errors due to the draft nature of the sequence. A summary of all regions more than 25 kb and a comparison to previously published data is shown in Table S2.

### Experimental Validation

**Figure 3:**
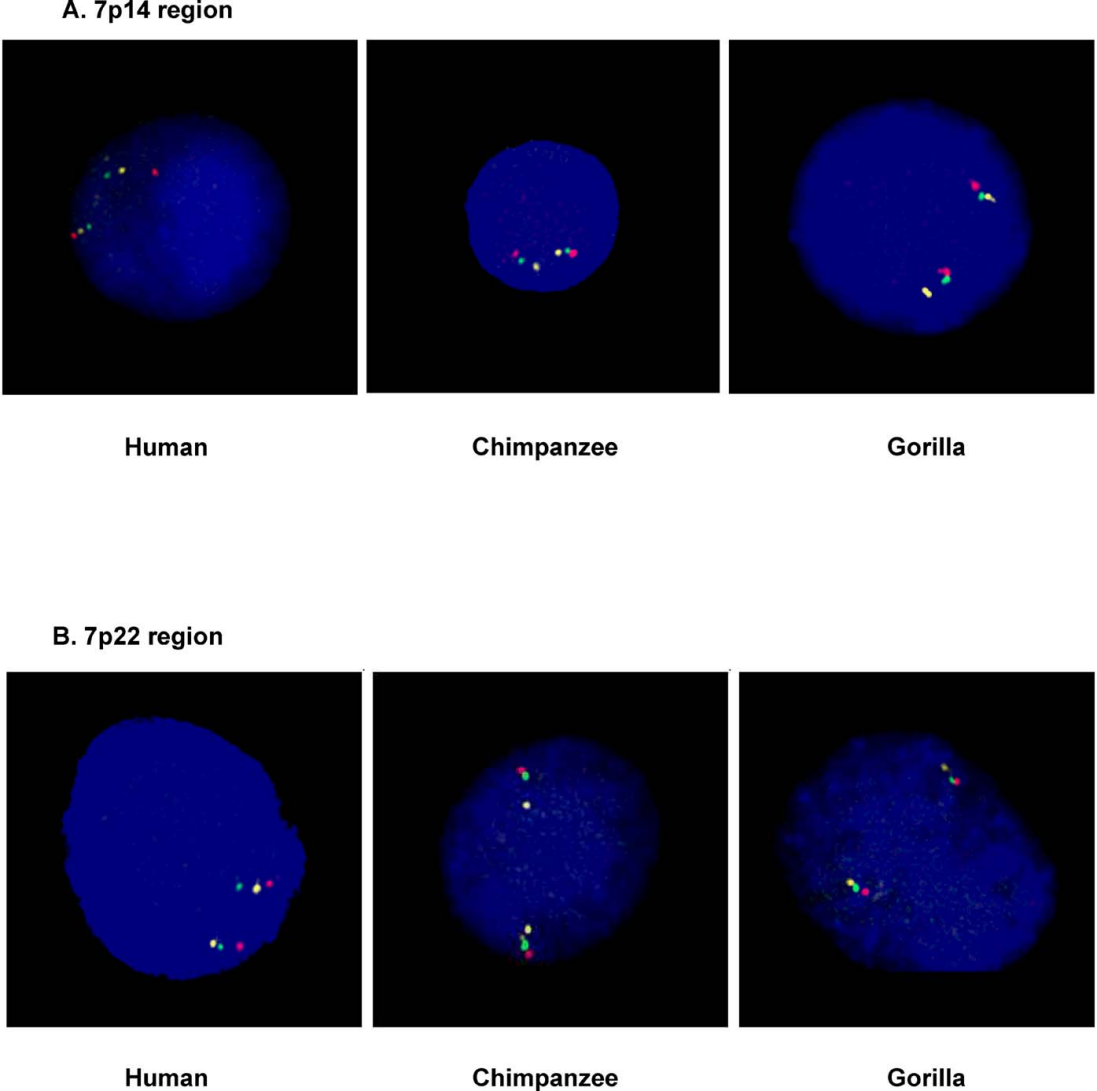
FISH Confirmation of Inversions (A) Three-color interphase FISH targeting the largest novel inversion between human and chimpanzee identified in this study. The probe order based on the human assembly is RP11-91E16 (red), RP11-321C5 (yellow), and RP11-81F19 (green). The result for human interphase testing is shown to the left and shows the expected the probe order red-yellow-green. The result for chimpanzee and gorilla displays the inverted probe order, red-green-yellow, using identical probes. For this region, each of ten human controls showed the same probe order. (B) Results showing an interphase nucleus from a human control polymorphic for the 730-kb inversion at 7p22. The probe order is red-yellow-green in the human assembly, and red-green-yellow in the chimpanzee assembly. The probe order for gorilla matches that of the chimpanzee.

Twenty-seven regions were selected for experimental validation (Table 1). These included five inversions larger than 500 kb, as well as the two regions that differ between the two human Chromosome 7 assemblies. The remaining regions were chosen to represent inversions of varying length. The initial phase of the project involved only Chromosome 7, and the selection of regions chosen for experimental validation is therefore biased towards this chromosome. The five largest inversions, 4.3 Mb at 7p22, 1.4 Mb at 2p25, 730 kb at 7p22, 680 kb at 19q13, and 670 kb at 7p12, were examined using three-color interphase fluorescence in situ hybridization (FISH) with bacterial artificial chromosomes (BACs) and fosmid probes. FISH experiments were performed using cell lines from human, chimpanzee, and gorilla. Gorilla was included as an out-group to determine in which lineage the inversion event occurred. Four of the five regions investigated by FISH were confirmed to be inverted between human and chimpanzee (Table 1), while the chimpanzee assembly did not match our results for a 1.4-Mb region on Chromosome 2. In three cases, the orientation of the region in gorilla matched that of the chimpanzee, indicating that the inversion is specific to the human lineage. The 4.3-Mb inversion at 7p14 is the largest inversion in this dataset not previously described in literature (Figure 3A). The inversion is almost entirely contained within the 7p14.1 band on G-banded chromosomes, which may explain why it was not detected in previous cytogenetic studies.

**Table 1:**
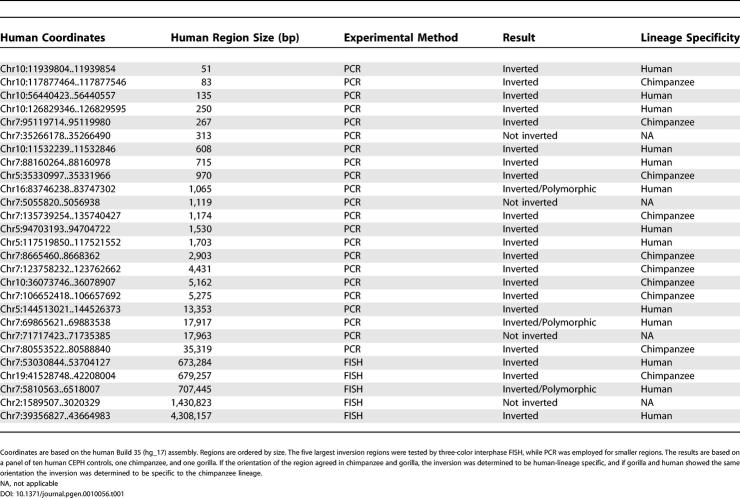
Regions That Were Tested Experimentally

The remaining 22 experimentally tested regions were investigated by PCR followed by DNA sequencing. Nineteen of these regions were shown to be inverted in chimpanzee compared to human. Of these, ten had the same orientation in gorilla and chimpanzee, while nine regions were the same in gorilla and human (Table 1). All regions that were inverted between human and chimpanzee were further tested in two additional chimpanzees, one additional gorilla, one orangutan, and one macaque. These results were also consistent, with the lower primates matching the orientation found in gorilla.

### Correlation with Genomic Features

Both inversions and copy number polymorphisms in the human genome show a strong correlation with regions containing segmental duplications [15,18,19,23]. Analyses of correlations between inversion regions and segmental duplications were, therefore, performed for all inversion regions in the human and chimpanzee genomes, respectively (Table S2). In both species there is a highly significant increase of segmental duplications around inversion breakpoints as compared to the genome-wide average (*p* < 0.0001 in both genomes). In the human genome, 75% of the 66 inversions more than 25 kb are flanked on one or both sides by segmental duplications. For 13 regions the flanking duplications are highly identical (96.6% average identity) and nine of these regions are of inverted orientation, which may explain the mechanism by which the inversion occurred.

Of the 1,576 putative inversion regions we detected computationally, 151 overlap RefSeq genes in the human genome assembly (Table S5), and 39 of these have one or more genes entirely contained within the inversion segment. Moreover, 83 inversions are contained within a gene (Table S6), and the remaining 29 regions have a breakpoint that intersects a gene, prioritizing them as candidates for biological and evolutionary studies.

### Identification of Human Polymorphic Inversions

**Figure 4:**
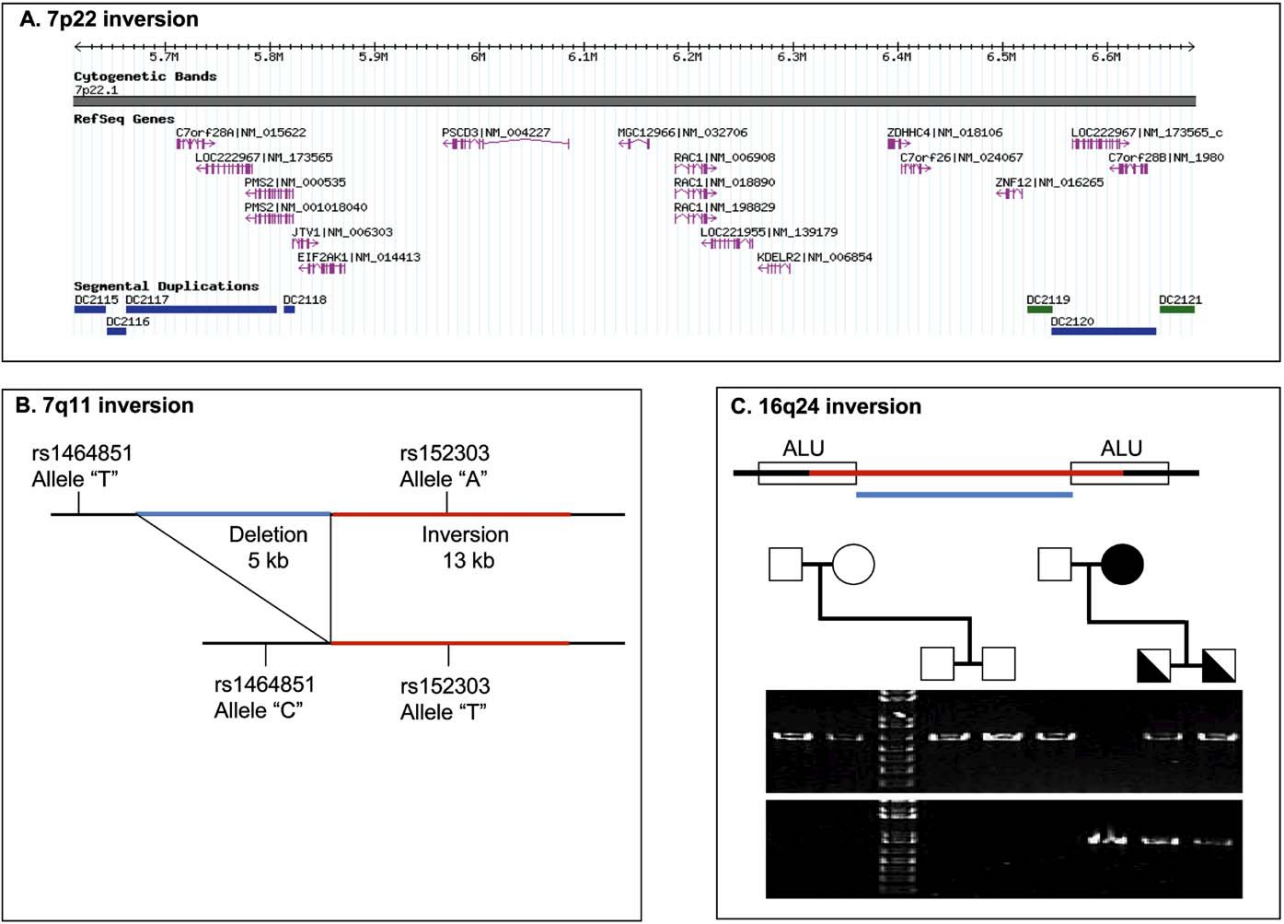
Overview of Polymorphic Regions (A) Overview of the region at 7p22 harbouring a 730-kb inversion variant. Each side of the inversion is flanked by highly identical segmental duplications of inverted orientation extending for ~100 kb with an average identity of 99%. Blue bars indicate that the duplications are intra-chromosomal, while green bars harbour both intra- and inter-chromosomal duplications. It is currently not clear exactly where within these segmental duplications the breakpoints occur. The region is comparatively gene-rich and provides an interesting target for diseases with linkage to this region. (B) Inversion polymorphism at 7q11. The inversion (shown in red) also led to a deletion of 5 kb (blue). The inversion and deletion variant is now the major allele. Two SNPs in perfect linkage disequilibrium (LD) with the inversion are also shown. There are no genes overlapping this inversion variant. (C) Inversion polymorphism at 16q24. This 1-kb inversion may have been induced by the flanking ALU repeats. The inversion indicated by the net alignment between human and chimpanzee is shown in blue. Experimental data show that the actual inversion (red) is approximately 400 bp longer than indicated by the net alignment. PCR results for two CEPH families are shown to the right. The top PCR was designed for the Build 35 assembly (652 bp) and the lower PCR was designed for the chimpanzee sequence (900 bp). The variant is inherited and shows the expected pattern of inheritance.

To confirm the orientation in human samples, all 23 experimentally validated inversions between human and chimpanzee were interrogated in ten unrelated individuals from the Centre d'etude du polymorphisme humain (CEPH) collection. The results for 20 of the 23 regions confirmed the orientation found in the initial human cell line. Three regions, however, were discovered to be polymorphic, with one allele matching the human assembly and the other allele matching the chimpanzee assembly. The first region, a 730- kb interval at human 7p22, was identified by interphase FISH, and two out of 20 individuals (10%) were found to be heterozygous (Figure 3B). The region is flanked on both sides by segmental duplications of high-sequence identity (Figure 4A). Detailed sequence analysis of these segmental duplications show several independent elements with both intra- and inter-chromosomal distribution patterns. One of these segmental duplications is present as a pair of duplicons on each side of the inverted region. The duplicons are of inverted orientation and extend for ~100 kb with an average sequence identity of 99%. The flanking duplications may be an indication that this inversion is a recurrent variant. In order to establish whether the inversion was a de novo event, both parents of one of the inversion carriers were tested. The results show that the variant was inherited from the mother, who was also a carrier for the inversion. The inversion region encompasses more than 15 RefSeq genes, including the *PMS2* gene, known to be involved in colorectal cancer [24].

The second inversion polymorphism, a 13-kb fragment at 7q11, corresponds to one of the regions that was also found to differ between the two human Chromosome 7 assemblies. The region is approximately 18 kb in size in the chimpanzee assembly, 18 kb in the National Center for Biotechnology assembly, and 13 kb in the CRA_TCAGchr7v2 assembly. The difference between the human assemblies is a 13-kb inversion associated with a 5-kb deletion (Figure 4B). There are no annotated genes in this region. Of the ten individuals tested initially, four were found to be homozygous for the inversion and deletion 13-kb region. The variant was found to be stably inherited as a polymorphism in a three-generation pedigree. Upon examination of the block pattern in the HapMap analysis [25] of this region, it was found that linkage disequilibrium with adjacent markers was very high. Eight CEPH samples that are part of the HapMap sample were then chosen based on their genotypes for five single nucleotide polymorphism (SNP) markers (rs1464853, rs1464851, rs1525303, rs1525287, and rs1568868) overlapping or flanking the inversion variant. In this small sample there was perfect linkage disequilibrium between the inversion variant and three SNP markers (Table 2). In fact, in the CEPH HapMap samples, marker rs152303, which is located within the inverted region, acts as a perfect surrogate marker for the inversion. Using the HapMap data we estimated the allele frequency for the inversion variant in CEPH samples of European ancestry (Table 3). The data show that the minor allele (18-kb allele, 30% frequency) matches the orientation of the chimpanzee genome and is represented in the National Center for Biotechnology Build 35 assembly, while the major allele (13-kb inversion and deletion allele) is represented in the CRA_TCAGchr7v2 assembly.

**Table 2:**
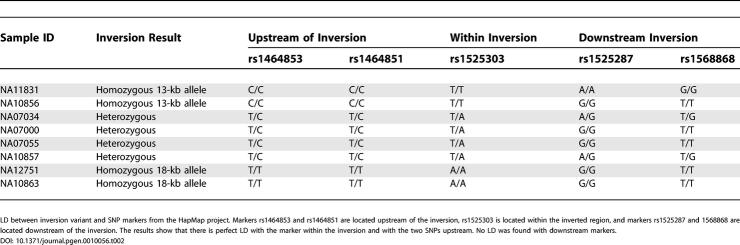
LD with 18-kb Inversion and Deletion Variant

**Table 3:**
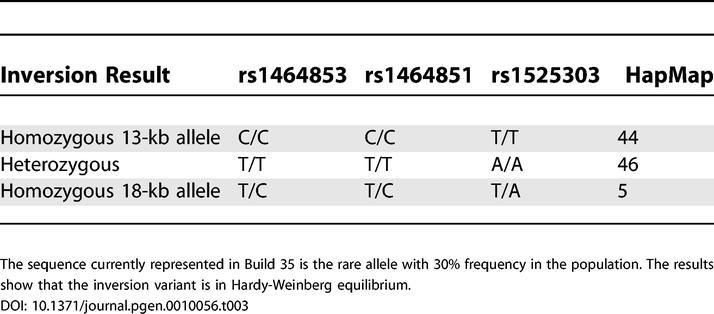
Frequency of the Inversion in the CEPH HapMap Samples Based on LD with Surrounding Markers

The third inversion polymorphism example is a 1,065-bp fragment located on Chromosome 16q24 (Figure 4C), with a minor allele frequency of 48% in 12 CEPH controls. The breakpoints occur within *Alu* repeats flanking the inversion on both sides. In this case the inversion was found to be 403 bp longer than indicated by the net alignment between human and chimpanzee. There are no genes overlapping this inversion and we found no evidence for linkage disequilibrium between surrounding markers in the HapMap data.

## Discussion

We describe the first comprehensive high-resolution study of inversions in recent primate evolution. With our experimentally verified data alone we more than double the catalogue of inversions that exist between the human and chimpanzee genomes. Importantly, we also identify another 1,549 putative inversion regions that can now be assessed experimentally. Taken together, our data indicate that inversions have been a frequent rearrangement in the evolution of the human genome and, as such, find that more than half of the experimentally validated inversions are specific to the human lineage. These results, in conjunction with the recent data from Tuzun et al., indicate that polymorphic inversions along chromosomes are common in the human population.

One of the limitations of the present study is that the chimpanzee assembly is currently a low-coverage draft sequence. This decreases the accuracy of the genome alignments. We therefore had to apply rigorous filtering criteria to reduce the vast number of false-positive inversion alignments. With a starting set of more than 6,000 regions from the raw alignment, only 1,576 regions remained after filtering. A separate source of chimpanzee genomic sequence is available through the fully sequenced BACs, which are currently not incorporated in the chimpanzee draft assembly. Analyses of the overlap between our computationally predicted regions and the BAC sequences supported 11 out of 60 regions that could be unambiguously mapped. As this analysis indicates, there is still a large fraction of false-positive inversions among these 1,576 regions. Some regions are also inherently difficult to assemble properly. One example is the 1.4-Mb region on Chromosome 2, where the chimpanzee assembly orientation was not confirmed by our FISH analysis during experimental validation. The region is flanked by gaps in both the human and chimpanzee assemblies, and we note that the orientation in the human assembly for this region was inverted between the two most recent assemblies of the human genome. With the currently available draft assembly for the chimpanzee, it is difficult to obtain a dataset devoid of false-positive inverted alignments. It is important to point out that the computational analysis provides a list of putative regions that are relevant for further studies and not a finalized list of inversion between the human and chimpanzee genomes. The release of the complete chimpanzee sequence in the future will greatly facilitate this type of approach and allow for a more accurate and complete identification of structural variation between the human and chimpanzee genomes.

The putative inversions are distributed throughout the genome with no obvious bias for specific regions of the chromosomes. A large fraction of the putative inversions are smaller than 250 bp in length. It is important to point out that net alignment programs are more likely to randomly assign orientation to very short alignments and the over-representation of very small inversions may therefore reflect a higher false-positive rate. In order to try and address this we performed an analysis of the percent identity between human and chimpanzee sequences for each of the alignments (Table S1). The results show that there is a lower average identity for regions less than 1 kb in size (Figure S1). The percent identity can be used as a quality measure for the alignments.

Our results show that there is a significant correlation between inversion events and segmental duplications. This was to be expected as segmental duplications have been shown to be associated both with copy number changes between human and chimpanzee [9,10] and with copy number variants in the human population [15,18,19]. This correlation is important for our understanding of the mechanisms underlying structural variation in the human genome. We also observe that the breakpoint of the 730-kb inversion on 7p22, which was found to be polymorphic in the human population, maps to exactly the same region as the breakpoint for the pericentric inversion that occurred after the divergence of higher primates from the orangutan [26]. This further supports the notion that chromosomal breakpoints have been reused throughout mammalian evolution [27].

The most interesting finding in this study is that three of the inversions were identified to be polymorphic in the human population. It would be expected that a certain fraction of the differences found between the human and chimpanzee assemblies are polymorphic in one of the two species, but perhaps not to the extent (13%) observed in this study. However, our selection of regions chosen for experimental validation was biased in that it included regions found to differ between the two human assemblies for Chromosome 7, thus increasing the chance to find regions that are polymorphic in the human population. Prior to the recent results by Tuzun et al., only a handful of inversions in the human genome were described (see The Database of Genomic Variants; http://projects.tcag.ca/variation/).

Characterization of inversion variants is important as they can instigate illegitimate recombination events leading to chromosome deletion in the off-spring of carriers. For example, a significantly higher incidence of inversions has been found in parents of patients in some microdeletion syndromes, including Williams-Beuren syndrome [28], Angelman syndrome[29], and Sotos syndrome [30]. Inversion may also act as suppressors of recombination, as was recently shown for a 900-kb inversion polymorphism on human Chromosome 17, which was also found to be positively selected for in several European populations [21]. Population studies are required to determine if the inversion polymorphisms identified here are recurrent or have increased in frequency after a single mutation event. The inversion and deletion polymorphism on 7q11 is unlikely to be a recurrent event as it is in perfect linkage disequilibrium with surrounding markers on a specific haplotype background.

In conclusion, we show that there have been a substantial number of inversion rearrangements in the human genome since the divergence from the chimpanzee. This finding indicates that inversion variants are likely to be abundant in the human genome, and this notion is further supported by the fact that three of the regions investigated in detail in this study were polymorphic in unrelated human samples. Many previously identified inversion variants have been linked to susceptibility to disease or increased risk for disease (usually via microdeletion) in the off-spring. Further studies aimed at understanding the impact the inversions identified in this study may have on gene expression and disease are now possible and are under way.

## Materials and Methods

### Identification of putative inversions.

The net human and chimpanzee alignments were downloaded from the University of California at Santa Cruz Web site (http://genome.ucsc.edu/). The net alignments were derived from BLASTZ alignments [31] generated by comparing the November, 2003 chimpanzee (panTro1) genome assembly and the May, 2004 (hg17) human genome assembly (http://genome.ucsc.edu/cgi-bin/hgTrackUi?hgsid=59218717&g=netPanTro1). The dataset was filtered to extract significant matches found to represent putative inversions. Matches to random chromosomes were removed, and only matches to syntenic chromosomes were maintained [8]. To reduce the number of false-positives, only those alignments better than or equal to level three were kept. This would preclude identification of inversions within inversions, but was required to reduce the number of potential artefacts. All inversion sequences were lower-case masked for highly repetitive elements by RepeatMasker (A.F. Smit and P. Green, unpublished data), and segmental duplications were downloaded from the human genome segmental duplication database (http://projects.tcag.ca/humandup) [32]. Net alignments with repeats or duplications that comprised greater than 90% of the inverted sequence were removed. The best reciprocal chain alignments from UCSC were obtained (panTro1.rbest.chain) to further refine the dataset to obtain the best set of inversions. In-house Perl scripts were developed to construct the rbest gapped alignment and this was used to filter out additional non-syntenic matches. The analysis of sequence identity for inverted regions was calculated as percent match, defined as the number of matching nucleotides within the inversion divided by the length of the alignment excluding insertions and deletions. To visualize the location and distribution of inversions, the data were converted and displayed using the publicly available visualization tool, GenomePixelizer [33] (http://www.atgc.org/GenomePixelizer/GenomePixelizer_Welcome.html).

### Correlation with segmental duplications and genes.

To determine the association between large inversions and flanking segmental duplications, the proximal and distal breakpoints of the putative inversions larger than 25 kb were scanned for the presence of segmental duplications [32]. A window of 25 kb (± 12.5 kb from each breakpoint) was examined for the presence or absence of duplications in the human and chimpanzee genomes (http://projects.tcag.ca/xenodup). These results were then compared with the genome-wide average derived from all 25-kb windows in the genome. A chi squared test was performed to determine the significance of the relationship.

The association between inversions and genes was examined to determine if any genes may have been interrupted by an inversion event. The current RefSeq dataset was downloaded from the UCSC Web site. In-house Perl scripts were developed to compare the location of genes and inversions. Three classes of relationships were described; genes spanning one inversion breakpoint, genes spanning both inversion breakpoints, and genes contained within an inversion.

We obtained the November, 2003 chimpanzee genome assembly through the UCSC Human Genome Browser. All chromosome sequences were lower-case masked for highly repetitive elements by RepeatMasker (A.F. Smit and P. Green, unpublished data). Each of the 26 masked chromosome sequences (including one unmapped chromosome sequence “ChrUn”) was compared against itself by chromosome-wide megaBLAST to detect intra-chromosomal segmental duplications. All possible pair-wise comparisons with each of the other 25 chromosomes were performed to detect inter-chromosomal segmental duplications. All BLAST results were subsequently filtered to eliminate low-quality and fragmented alignments according to methods previously described [32]. The data are displayed in the non-human genome segmental duplication database (http://projects.tcag.ca/xenodup).

### Analysis of chimpanzee BAC clones.

To obtain additional support for putative inversions, fully sequenced chimpanzee BAC clones were mapped to the human genome assembly by BLAST, and those that overlapped breakpoints of inversions were detected. A sequence comparison of the chimpanzee clone and human DNA at the inversion breakpoint was performed. The inversion sequence along with 1 kb of flanking DNA sequence from the human genome was compared by BLAST with the entire chimpanzee clone and each segment (flanking proximal, inversion, flanking distal) was scored as + or − in orientation. Therefore a +/−/+ or −/+/− match was taken to confirm the inversion. Entries for which a significant match was not found for any of the three segments were analyzed manually.

### PCR and sequencing.

A total of three oligonucleotide primers were designed for each inversion region with one primer within the inversion region based on the human orientation, one primer within the inversion region based on the chimpanzee orientation, and one primer outside the inversion region. All primers were optimized using a gradient hybridization temperature from 56–61 °C. The PCR cycling conditions were 95 °C for 5 min followed by 38 cycles of (95 °C 15 s, optimized hybridization temperature 30 s, 72 °C 60 s per kb product length) and a final extension 72 °C for 5 min. PCR primers and optimized hybridization temperatures are available upon request.

### Fluorescence in situ hybridization.

FISH was used to score inversion both between humans and chimpanzees, as well as for scoring of inversion polymorphism in humans. Each region was interrogated using three- color interphase FISH with two probes within the putative inversion region and a reference probe outside. BAC clones were used as probes for four of the region, while fosmid clones were used for the 7q12 region. Each clone was first tested on DAPI stained metaphase chromosomes to ensure that each probe mapped uniquely to the correct chromosomal location. At least 100 interphase nuclei were scored for each probe set. The polymorphic region on 7p22 was further validated with a set of independent probes. The probes used for FISH are shown in Table S7. Interphase nuclei were hybridized as previously described [28].

### Cell lines and DNA samples.

Human CEPH lymphocyte cell lines were obtained from Coriell Cell Repositories (Coriell, Camden, New Jersey, United States) and primate lymphocyte cell and fibroblast lines were obtained from The European Collection of Cell Cultures. Genomic DNA was extracted from cell lines. The ten CEPH individuals used for FISH screening were: GM10859, GM7057, GM06990, GM10858, GM10832, GM13114, GM13180, GM13181, GM10835, and GM10834. The chimpanzee and gorilla cell lines used for FISH analysis were ECACC cell lines #89072704 and #89072703. DNA from healthy controls of European ancestry was used for PCR based assays.

## Supporting Information

Figure S1 Sequence Identity for Putative Inversion Distribution of percent match between human and chimpanzee sequences for inverted regions. This distribution indicates that regions less than 1 kb in size are more likely to contain false-positive inversions. The percent match for each region is shown is Table S1 and can be viewed as a quality measure for the underlying alignment.
(28 KB PDF)

Table S1 All Putative 1,576 Inversion Regions between the Human and Chimpanzee Genomes (116 KB PDF)

Table S2 Inversions More than 25 kb (89 KB XLS)

Table S3 Previously Published Inversions between the Human and Chimpanzee Genomes (24 KB XLS)

Table S4 Comparison to Previously Published Inversions (24 KB XLS)

Table S5 Inversions Overlapping Genes (20 KB XLS)

Table S6 Inversions Contained within Genes (28 KB XLS)

Table S7 Probes Used for FISH Experiments (19 KB XLS)

## Abbreviations

BAC: bacterial artificial chromosome
bp: base pair
CEPH: Centre d'etude du polymorphisme humain
FISH: fluorescence in situ hybridization
kb: kilobase
LD: linkage disequilibrium
Mb: mega-base
