# Supplementary figures and images for: Discovery of Human Inversion Polymorphisms by Comparative Analysis of Human and Chimpanzee DNA Sequence Assemblies
Source: PLoS Genet. 2005 Oct 28;1(4):e56. doi: 10.1371/journal.pgen.0010056 (PMC1270012; doi:10.1371/journal.pgen.0010056)

Figure S1. Sequence identity for putative inversions

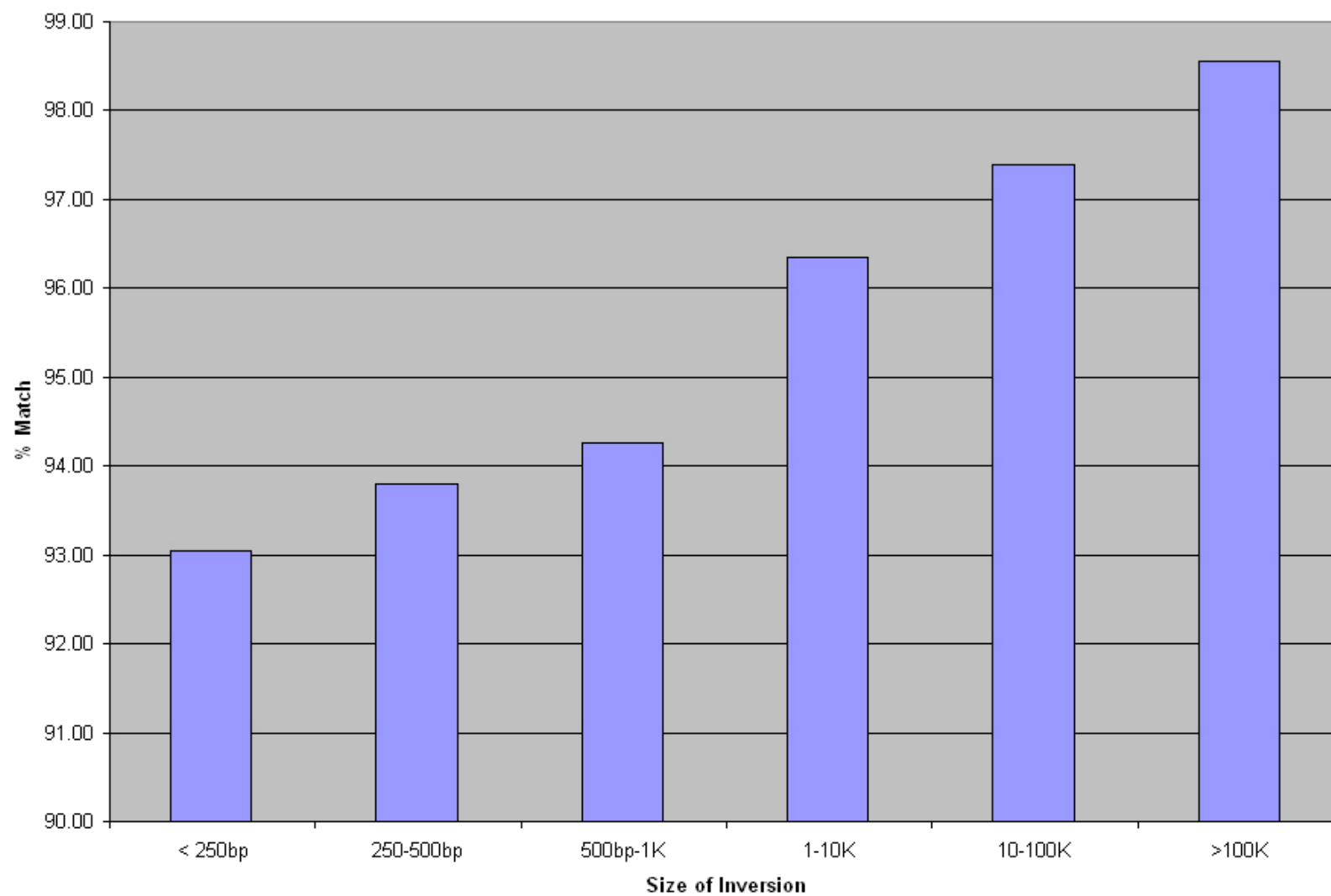

Supplement: Figure S1 — Distribution of percent match between human and chimpanzee sequences for inverted regions. This distribution indicates that regions less than 1 kb in size are more likely to contain false-positive inversions. The percent match for each region is shown is Table S1 and can be viewed as a quality measure for the underlying alignment. (28 KB PDF) [file pgen.0010056.sg001.pdf]
