# Supplementary material for: Discovery of Human Inversion Polymorphisms by Comparative Analysis of Human and Chimpanzee DNA Sequence Assemblies
Source: PLoS Genet. 2005 Oct 28;1(4):e56. doi: 10.1371/journal.pgen.0010056 (PMC1270012; doi:10.1371/journal.pgen.0010056)
Supplement: Table S1 — (116 KB PDF) [file pgen.0010056.st001.pdf]

Human Chr	Human Start	Human Stop	Chimp Chr	Chimp Start	Chimp Stop	Human Length	Chimp Length	% Dup Coverage	% Repeat Coverage	% Match
chr5	34249652	95947182	chr4	19219894	82959708	61697531	63739815	4	45	98.78
chr4	44652593	65134026	chr3	81507847	102312984	20481434	20805138	1	43	98.57
chr4	73407826	86315437	chr3	47358490	60327796	12907612	12969307	0	48	98.79
chr18	102544	10913022	chr17	1882009	12697899	10810479	10815891	0	47	98.63
chr15	18313193	26244831	chr16	20542252	28367385	7931639	7825134	34	50	98.23
chr5	21921269	26652011	chr4	90802394	95501268	4730743	4698875	4	50	98.45
chr4	65139017	69862541	chr3	76993784	81502838	4723525	4509055	11	51	98.5
chr7	40653710	43664983	chr6	40575221	43598946	3011274	3023726	0	44	98.87
chr4	70542959	73403379	chr3	60332218	76524041	2860421	16191824	2	47	98.59
chr5	26675757	29483971	chr4	87964410	90786579	2808215	2822170	0	50	98.42
chr5	29491092	32038443	chr4	85333796	87957541	2547352	2623746	0	48	98.52
chr5	18589078	20807514	chr4	99462132	101743160	2218437	2281029	3	47	98.58
chr1	109938476	111948702	chr1	110317888	112543951	2010227	2226064	1	46	98.79
chr15	26996736	28489097	chr16	18428715	20042977	1492362	1614263	22	49	98.61
chr2	1589507	3020329	chr12	1618753	3079857	1430823	1461105	0	35	98.45
chr1	111949406	113371599	chr1	108881401	110316525	1422194	1435125	0	49	98.88
chr7	39358321	40652615	chr6	43599928	45161662	1294295	1561735	4	50	98.92
chr7	5810563	6518007	chr6	6281602	6992559	707445	710958	1	55	98.61
chr19	41528748	42208004	chr20	38571492	39250051	679257	678560	3	65	98.62
chr5	98949085	99410215	chr4	104683812	105147879	461131	464068	1	49	98.58
chr11	49329112	49785709	chr9	90042647	90562701	456598	520055	90	50	97.05
chr5	21558880	21920651	chr4	45150549	45509920	361772	359372	0	46	98.54
chr7	53351230	53704127	chr6	54721836	55078142	352898	356307	0	55	98.5
chr9	43099880	43447222	chr11	40032800	40562197	347343	529398	82	40	97.9
chr7	53030844	53337047	chr6	55092324	55400709	306204	308386	0	48	98.36
chr19	16750522	17003347	chr20	17983388	18240445	252826	257058	0	59	98.35
chr1	113410402	113655554	chr1	112590603	112886082	245153	295480	0	54	98.73
chr17	57421435	57660951	chr19	47477836	47717935	239517	240100	12	57	98.89
chr6	61989990	62185125	chr5	62559950	62769928	195136	209979	35	71	97.84
chr10	51907296	52095219	chr8	52569883	52758942	187924	189060	5	40	99.02
chr1	202704365	202875288	chr1	186160249	186330610	170924	170362	16	48	98.66
chr17	57693605	57850491	chr19	47311420	47470565	156887	159146	27	53	98.67
chr16	28390711	28545637	chr18	29110008	29273443	154927	163436	11	58	98.5
chr10	51598875	51753561	chr8	52912725	53089551	154687	176827	46	46	98.83
chr7	142801072	142942441	chr6	145554696	145702226	141370	147531	89	53	98.55
chr19	55135022	55260077	chr20	53105203	53230106	125056	124904	0	59	98.53
chr19	42305639	42425485	chr20	38338869	38473811	119847	134943	0	56	98.49
chrX	71748845	71860873	chrX	74138760	74248141	112029	109382	0	61	99.24
chr15	28757711	28855705	chr16	29273235	29379413	97995	106179	78	56	97.75
chr19	20312197	20398955	chr20	21662741	21690020	86759	27280	0	77	88.49
chr4	70067550	70142281	chr3	76744159	76818784	74732	74626	22	58	98.5
chrX	138877264	138950857	chrX	143729418	143804185	73594	74768	0	55	98.96
chr22	20075270	20146044	chr23	20128104	20201311	70775	73208	78	45	98.66
chr4	70470185	70538225	chr3	76528781	76597745	68041	68965	36	49	98.38
chr9	87810588	87878496	chr11	71829384	71893709	67909	64326	0	67	98.78
chrX	51344190	51410012	chrX	52327911	52395065	65823	67155	0	80	99.15
chr2	89905079	89970892	chr12	92024252	92118503	65814	94252	79	42	98.54
chr7	63698718	63762463	chr6	65466545	65522817	63746	56273	8	64	88.51
chr10	51806257	51866853	chr8	52799416	52860029	60597	60614	0	31	99.14
chr17	41673868	41733269	chr19	63397185	63465787	59402	68603	88	51	98.54
chr1	245123022	245181641	chr1	229037924	229095835	58620	57912	58	38	97.94
chr12	46134049	46191062	chr10	47104355	47158728	57014	54374	0	58	98.53

chr10	26910848	26967492	chr8	27544034	27953365	56645	409332	67	44	91.14
chrX	148454375	148509463	chrX	153671767	153727175	55089	55409	35	60	99.01
chr7	56532805	56587744	chr6	64229321	64287641	54940	58321	70	66	95.1
chrX	138814002	138863111	chrX	143818338	143867471	49110	49134	17	72	98.94
chrX	148353791	148402585	chrX	153781671	153841312	48795	59642	59	40	97.57
chr11	49823370	49871957	chr9	49376010	49406582	48588	30573	74	27	94.72
chr17	54956779	55003655	chr19	50169262	50216602	46877	47341	0	59	98.78
chr16	16613986	16659869	chr18	19070399	19113249	45884	42851	88	70	98.41
chr16	68667028	68711650	chr18	67338518	67534224	44623	195707	90	42	98.3
chr19	21609852	21650666	chr20	22641180	22704644	40815	63465	34	73	92.64
chr4	69881467	69921783	chr3	76967336	76974414	40317	7079	0	76	98.14
chr19	44744970	44783796	chr20	41790122	41825382	38827	35261	0	55	98.24
chrX	153083454	153120937	chrX	158419857	158459689	37484	39833	30	16	98.93
chr7	71641035	71678158	chr6	67690928	67735494	37124	44567	37	84	98.53
chr10	38360042	38395653	chr8	42945019	42989834	35612	44816	52	74	96.02
chr7	80553522	80588840	chr6	81781455	81816825	35319	35371	0	41	98.89
chrX	51746841	51780730	chrX	52830687	52858631	33890	27945	81	55	99.28
chr9	85570782	85603845	chr11	67670841	67703968	33064	33128	37	60	98.96
chr8	39420536	39451620	chr7	40814662	40844118	31085	29457	0	63	83.33
chr16	20346250	20377247	chr18	20925778	20961180	30998	35403	86	63	97.93
chr4	69927072	69955942	chr3	76930351	76962050	28871	31700	33	62	98.51
chr12	130292239	130321107	chr10	133831465	133869069	28869	37605	87	30	96.06
chr1	142408093	142436732	chr1	113013969	113042615	28640	28647	3	32	99.09
chr2	95612830	95641389	chr12	98601718	98624749	28560	23032	63	37	99.02
chrX	51245702	51273574	chrX	52468686	52498511	27873	29826	83	67	98.96
chr19	56380306	56407909	chr20	54051621	54079850	27604	28230	0	47	86.01
chr2	95584615	95612009	chr12	98625570	98652750	27395	27181	82	29	99.12
chr8	47896328	47923452	chr7	49477431	49498131	27125	20701	0	85	95.12
chr1	142441814	142468082	chr1	112982531	113008920	26269	26390	0	56	99.01
chr19	20640153	20665417	chr20	21485215	21510456	25265	25242	0	77	98.27
chr2	95642238	95667415	chr12	98575492	98600867	25178	25376	0	33	99.03
chrX	70756169	70780706	chrX	72972808	72997587	24538	24780	78	38	98.99
chrX	52727950	52751300	chrX	53772528	53793430	23351	20903	44	42	98.87
chrX	148413013	148436299	chrX	153746228	153771082	23287	24855	0	46	98.68
chr1	142366261	142389112	chr1	113063598	113086593	22852	22996	74	20	98.93
chr10	46456036	46478424	chr8	47442764	47467450	22389	24687	0	46	98.75
chr1	113376859	113399189	chr1	112897292	112921391	22331	24100	0	33	99.04
chr11	131991442	132013494	chr9	134162932	134185796	22053	22865	0	35	98.59
chrX	46567039	46586760	chrX	48143503	48163298	19722	19796	0	53	99.21
chrX	134513047	134532662	chrX	139362150	139374930	19616	12781	36	85	90.18
chrX	70357459	70376725	chrX	72625218	72645640	19267	20423	0	59	98.58
chr19	46084464	46103714	chr20	43232837	43257402	19251	24566	87	55	97.87
chrX	51299840	51319066	chrX	52420189	52439697	19227	19509	8	62	99.16
chr5	21410252	21429294	chr4	95992994	96012082	19043	19089	85	47	98.81
chr2	181457577	181476067	chr13	71255064	71273715	18491	18652	0	50	98.35
chr11	1649113	1667300	chr9	1780384	1798234	18188	17851	34	59	97.97
chrX	103057725	103075883	chrX	106442722	106460808	18159	18087	0	49	98.47
chr7	71717423	71735385	chr6	67633150	67651204	17963	18055	65	75	98.79
chrX	70402015	70419889	chrX	72552442	72572875	17875	20434	0	66	99.05
chr8	16188127	16205890	chr7	16940697	16961590	17764	20894	0	46	97.83
chr4	147242947	147260093	chr3	164494747	164511914	17147	17168	0	80	98.41
chr19	55270935	55287834	chr20	53242244	53258101	16900	15858	40	81	96.91
chr1	105075590	105092224	chr1	104130057	104146363	16635	16307	0	69	97.07

chr9	112932144	112948528	chr11	97479833	97495489	16385	15657	0	36	98.33
chr4	69980747	69996651	chr3	76890067	76905858	15905	15792	0	79	98.55
chr19	20682802	20698595	chr20	21451744	21467653	15794	15910	0	64	98.15
chrX	105311594	105327277	chrX	108897064	108911994	15684	14931	74	63	98.74
chr4	133410549	133425660	chr3	150399225	150414438	15112	15214	0	74	98.65
chr1	32551140	32565896	chr1	33301125	33316277	14757	15153	0	64	98.17
chr1	109923787	109938475	chr1	112497044	112511796	14689	14753	72	44	97.71
chr9	123818209	123832871	chr11	108557916	108572680	14663	14765	0	55	98.75
chrX	148523838	148538366	chrX	153645814	153663264	14529	17451	84	45	98.52
chr19	20415247	20429222	chr20	21122176	21136112	13976	13937	0	63	89.74
chrX	51230041	51243942	chrX	52500113	52513970	13902	13858	0	78	99.08
chr4	70408774	70422514	chr3	76641524	76655605	13741	14082	87	61	97.92
chr5	144513021	144526373	chr4	151189848	151203168	13353	13321	0	42	98.82
chr4	70422628	70435659	chr3	76621260	76634279	13032	13020	78	86	98.41
chr8	48378817	48391668	chr7	49927693	49940672	12852	12980	22	85	98.9
chr1	143744839	143757552	chr1	127177184	127190652	12714	13469	6	51	95.66
chr15	100145309	100157947	chr16	99231735	99243659	12639	11925	53	34	98.3
chr1	20597128	20609738	chr1	20894566	20907019	12611	12454	0	66	98.54
chrX	149242145	149254719	chrX	154471926	154484543	12575	12618	0	29	98.3
chrY	16439375	16451747	chrY	17094809	17111452	12373	16644	44	58	97.72
chr3	170088600	170100757	chr2	173567686	173580049	12158	12364	0	59	98.21
chr12	130402005	130413682	chr10	133441868	133451600	11678	9733	18	57	92.47
chr11	1882620	1892956	chr9	2086470	2096796	10337	10327	0	71	98.14
chrX	145589216	145599160	chrX	150782653	150788554	9945	5902	0	76	98.2
chr21	40320324	40329742	chr22	40364021	40373719	9419	9699	0	59	97.32
chr14	23327740	23337016	chr15	22270661	22280363	9277	9703	0	79	98.35
chrX	70734203	70743415	chrX	73010767	73019521	9213	8755	0	79	98.58
chr12	85744858	85754030	chr10	87868698	87877955	9173	9258	0	35	98.9
chr1	113669596	113678640	chr1	112567480	112576558	9045	9079	0	37	99.06
chr3	50902683	50911200	chr2	52485099	52493604	8518	8506	0	59	97.81
chr4	31374744	31383231	chr3	31972192	31983043	8488	10852	0	35	99.04
chr4	133402241	133410548	chr3	150390869	150399111	8308	8243	0	70	98.88
chr19	41514672	41522788	chr20	39250152	39265706	8117	15555	0	63	99.1
chr4	89204343	89212421	chr3	105199787	105207803	8079	8017	0	80	98.03
chr19	42426358	42434425	chr20	38329947	38337996	8068	8050	0	67	98.64
chr8	50529355	50537114	chr7	52115939	52123686	7760	7748	0	30	98.48
chr14	35454226	35461892	chr15	34781017	34788613	7667	7597	0	69	98.44
chr6	93505644	93513309	chr5	95250727	95258373	7666	7647	0	39	98.3
chr1	26649408	26657054	chr1	27336701	27344650	7647	7950	0	64	97.93
chr16	54379200	54386825	chr18	48474324	48481793	7626	7470	90	47	98.44
chrX	100673638	100681250	chrX	103919573	103928360	7613	8788	0	0	99.33
chr17	27445700	27453200	chr19	30813552	30840797	7501	27246	25	40	98.56
chr1	89526899	89534178	chr1	88402719	88408721	7280	6003	0	53	85.71
chr1	113689399	113696451	chr1	112549219	112556726	7053	7508	0	39	99.12
chr19	43960157	43967092	chr20	40889975	40899409	6936	9435	0	67	98.57
chr1	2523902	2530769	chr1	2441638	2449022	6868	7385	0	0	97.99
chr5	178996023	179002842	chr4	186527453	186533885	6820	6433	0	59	98.43
chr17	23183532	23189997	chr19	26512640	26519113	6466	6474	0	86	98.92
chr3	850688	857030	chr2	870711	877143	6343	6433	0	24	98.04
chrY	20591702	20597939	chrY	21653711	21660689	6238	6979	0	52	98.27
chrX	54951041	54957226	chrX	56217249	56223146	6186	5898	0	69	99.01
chr9	114294735	114300890	chr11	98829838	98836015	6156	6178	0	82	98.7
chr4	138000890	138007037	chr3	155178372	155190586	6148	12215	0	88	98.19

chr5	105928549	105934652	chr4	111817053	111823176	6104	6124	0	35	98.49
chr2	234262677	234268659	chr13	124989518	124994252	5983	4735	0	36	96.9
chrX	72079036	72084951	chrX	74274927	74280847	5916	5921	77	30	98.58
chrX	55394598	55400449	chrX	56747050	56752905	5852	5856	0	10	99.49
chrX	136705671	136711436	chrX	141372842	141378601	5766	5760	0	54	99
chr8	145052802	145058531	chr7	148331376	148337105	5730	5730	0	80	98.48
chrX	153121561	153127182	chrX	158413497	158419235	5622	5739	0	24	98.82
chr13	81825546	81830859	chr14	64534970	64540282	5314	5313	0	77	98.89
chr10	27246312	27251604	chr8	27274800	27279852	5293	5053	0	57	92.55
chr10	36073746	36078907	chr8	36579599	36584794	5162	5196	0	36	98.41
chrX	48770140	48775268	chrX	50094857	50100018	5129	5162	0	67	98.99
chr14	39050969	39056010	chr15	38411245	38416290	5042	5046	0	47	98.52
chr3	167822181	167827208	chr2	171280093	171285134	5028	5042	0	50	97.87
chr14	63401631	63406567	chr15	62971561	62976464	4937	4904	0	45	98.2
chrX	44234471	44239382	chrX	45684191	45689106	4912	4916	0	79	96.19
chr11	123763149	123767999	chr9	125842835	125847683	4851	4849	0	65	98.55
chr3	195538179	195542939	chr2	199663960	199668731	4761	4772	0	51	97.68
chrX	101556657	101561388	chrX	104549605	104554465	4732	4861	0	61	99.17
chr4	166196247	166200976	chr3	183661120	183664065	4730	2946	10	66	87.27
chr3	160598737	160603447	chr2	163775240	163780006	4711	4767	0	72	97.41
chr19	41524055	41528747	chr20	39257186	39261914	4693	4729	0	51	98.68
chr5	101051689	101056216	chr4	119354501	119359034	4528	4534	0	34	98.96
chr7	39199429	39203939	chr6	45164706	45169239	4511	4534	0	17	98.43
chr7	123758232	123762662	chr6	126115211	126119626	4431	4416	0	50	98.34
chr4	134282344	134286773	chr3	151263251	151267665	4430	4415	0	88	98.91
chrX	153127822	153132095	chrX	158408556	158412857	4274	4302	0	14	99.01
chrX	75151491	75155615	chrX	77748642	77752774	4125	4133	0	38	99.2
chr21	38482159	38486280	chr22	39206777	39209844	4122	3068	0	48	83.85
chr19	51364962	51369039	chr20	48705923	48710207	4078	4285	0	60	89.28
chr5	105934680	105938726	chr4	111812991	111817025	4047	4035	0	39	96.57
chr15	86187948	86191937	chr16	87032683	87036699	3990	4017	0	26	98.17
chr19	43956080	43960021	chr20	40899545	40903498	3942	3954	42	46	98.8
chr10	5013948	5017874	chr8	5090510	5094429	3927	3920	0	67	98.16
chr2	8047586	8051501	chr12	8699866	8703776	3916	3911	0	12	97.82
chrY	8691566	8695410	chrY	8325109	8329805	3845	4697	0	30	85.88
chr19	48550463	48554156	chr20	45475602	45479255	3694	3654	0	28	96.61
chr16	73810467	73814151	chr18	68195013	68198582	3685	3570	0	58	96.54
chrX	145575616	145579299	chrX	150769617	150773299	3684	3683	0	63	98.45
chr4	70168481	70172151	chr3	76714187	76717852	3671	3666	0	80	98.85
chr10	124646783	124650417	chr8	126982797	126987635	3635	4839	0	13	84.91
chr6	30466076	30469665	chr5	30971525	30974268	3590	2744	0	61	80.5
chr2	138838732	138842320	chr13	27851068	27854662	3589	3595	0	79	98.8
chr9	17469668	17473208	chr11	17709896	17712904	3541	3009	0	50	98.42
chr11	13577770	13581277	chr9	13794964	13798125	3508	3162	0	50	88.91
chr19	20666654	20670128	chr20	21480510	21483978	3475	3469	0	76	98.14
chrX	47706775	47710211	chrX	49286126	49289563	3437	3438	0	39	98.84
chr16	54195836	54199127	chr18	48257868	48261286	3292	3419	0	80	98.06
chr10	124694369	124697641	chr8	126945253	126947267	3273	2015	0	84	85.79
chr19	42435131	42438387	chr20	38326012	38329272	3257	3261	46	79	98.09
chr20	45912024	45915219	chr21	49188064	49191757	3196	3694	0	63	87.48
chrX	55389764	55392909	chrX	56756131	56759249	3146	3119	0	11	99.09
chr22	22640606	22643598	chr23	22672458	22675453	2993	2996	0	20	99.2
chr6	161237604	161240514	chr5	165127993	165135560	2911	7568	0	23	85.67

chr7	8665460	8668362	chr6	9112677	9115597	2903	2921	0	56	97.55
chr4	43920018	43922902	chr3	46622261	46625266	2885	3006	0	55	98.65
chr4	190985452	190988265	chr3	209200281	209203094	2814	2814	0	27	97.51
chrX	145609880	145612689	chrX	150779596	150782468	2810	2873	0	71	93.94
chr9	135666186	135668942	chr11	118135996	118139262	2757	3267	71	6	92.12
chr3	43649892	43652606	chr2	44941914	44944653	2715	2740	0	48	98.82
chr14	24035952	24038656	chr15	22933309	22936028	2705	2720	0	47	97.56
chr19	23108855	23111530	chr20	22570306	22571883	2676	1578	0	67	85.43
chr4	181250393	181253025	chr3	199378116	199380745	2633	2630	0	23	98.66
chr19	11998329	12000934	chr20	12412138	12414745	2606	2608	0	71	99.04
chr1	2520571	2523162	chr1	2449762	2452393	2592	2632	0	8	98.72
chr12	41131550	41134133	chr10	41724125	41726731	2584	2607	0	52	97.86
chrX	71978314	71980895	chrX	74018696	74021284	2582	2589	0	39	99.25
chr5	177317719	177320282	chr4	184821613	184824244	2564	2632	0	19	96.47
chr6	69906942	69909500	chr5	71019770	71022258	2559	2489	0	56	98.55
chrX	53781803	53784266	chrX	55067585	55069340	2464	1756	0	68	94.72
chr10	93193325	93195766	chr8	94916473	94918921	2442	2449	0	29	99.14
chr12	121450986	121453340	chr10	124522273	124524645	2355	2373	0	88	96.15
chr3	54167662	54169999	chr2	55817738	55820077	2338	2340	0	58	98.55
chr6	107275246	107277570	chr5	109436576	109438923	2325	2348	0	49	98.15
chr4	70197928	70200236	chr3	76696390	76698658	2309	2269	0	35	98.38
chr2	119669804	119672106	chr13	5691849	5694536	2303	2688	0	2	97.7
chr21	17343291	17345441	chr22	17425394	17449830	2151	24437	0	41	98.92
chr15	43014785	43016933	chr16	42960648	42962818	2149	2171	0	62	96.58
chr1	142486281	142488390	chr1	112961468	112964330	2110	2863	0	64	98.67
chr1	20619586	20621685	chr1	20881595	20883691	2100	2097	0	73	98.52
chrX	17552667	17554760	chrX	18616109	18618932	2094	2824	0	0	96.83
chr15	35356147	35358235	chr16	35161576	35163671	2089	2096	0	78	97.56
chr17	18867887	18869894	chr19	19361772	19363349	2008	1578	33	37	98.42
chr1	20611728	20613661	chr1	20890637	20892572	1934	1936	0	86	98.6
chr19	51390639	51392571	chr20	48729825	48732485	1933	2661	0	0	89.97
chr1	228706837	228708743	chr1	212566406	212566832	1907	427	0	3	93.61
chr8	6142792	6144697	chr7	6356679	6358566	1906	1888	0	67	96.81
chr6	93513346	93515235	chr5	95248807	95250690	1890	1884	0	43	98.14
chr5	163306621	163308503	chr4	170366078	170367949	1883	1872	0	0	89.48
chrX	50725645	50727489	chrX	51727046	51728266	1845	1221	0	63	89.38
chr10	58276526	58278360	chr8	59191962	59193795	1835	1834	0	75	98.58
chr4	69877633	69879425	chr3	76976450	76978240	1793	1791	0	26	98.99
chr19	15646185	15647967	chr20	16454643	16456353	1783	1711	0	38	90.58
chrX	50616684	50618452	chrX	51836086	51837205	1769	1120	0	37	91.89
chr18	12050572	12052337	chr17	1862683	1864985	1766	2303	0	70	85.58
chr12	39673826	39675585	chr10	40258707	40260465	1760	1759	0	13	99.2
chr19	43967231	43968946	chr20	40888114	40889836	1716	1723	0	80	98.82
chr22	16986339	16988050	chr23	16912130	16913845	1712	1716	0	13	98.71
chr5	117519850	117521552	chr4	123722606	123724342	1703	1737	0	1	98.77
chr19	15662068	15663746	chr20	16440943	16442657	1679	1715	0	60	88.9
chr19	20583565	20585228	chr20	21565703	21567382	1664	1680	0	68	98.54
chr2	138836410	138838064	chr13	27855331	27856983	1655	1653	0	20	99.03
chr3	44513014	44514618	chr2	45877198	45878616	1605	1419	21	37	84.13
chr17	11790717	11792297	chr19	12142940	12144547	1581	1608	0	38	98.02
chrX	122657933	122659508	chrX	126789932	126791508	1576	1577	0	31	98.85
chr9	19154498	19156068	chr11	19434225	19435764	1571	1540	0	82	87.58
chr1	236481630	236483191	chr1	220405601	220406860	1562	1260	0	31	93.62

chr12	85743314	85744837	chr10	87877976	87879498	1524	1523	0	66	97.37
chr12	65800790	65802309	chr10	67370700	67372220	1520	1521	0	51	99.14
chr19	52997650	52999148	chr20	50375200	50376746	1499	1547	0	20	95.07
chrX	101977738	101979227	chrX	105492838	105495459	1490	2622	0	21	82.27
chr2	35422484	35423968	chr12	36837336	36838807	1485	1472	0	12	98.5
chrX	148436346	148437797	chrX	153751620	153753069	1452	1450	0	5	98.83
chr1	42500345	42501792	chr1	43470748	43472204	1448	1457	0	60	97.36
chr19	55132132	55133579	chr20	53231566	53233002	1448	1437	0	58	99.02
chr19	39847214	39848657	chr20	36322909	36324373	1444	1465	0	2	90.74
chr3	178384355	178385788	chr2	182107531	182108971	1434	1441	0	23	99.3
chr1	20593108	20594513	chr1	20910458	20911857	1406	1400	0	52	99.28
chr21	22313574	22314972	chr22	22481936	22483438	1399	1503	0	43	98.99
chr4	43049696	43051071	chr3	45740992	45742361	1376	1370	0	0	98.1
chr20	8780741	8782086	chr21	8669530	8670868	1346	1339	0	14	99.03
chr21	42433458	42434800	chr22	42562887	42564232	1343	1346	0	23	96.95
chrX	5996786	5998120	chrX	5963218	5964519	1335	1302	0	36	98.3
chr16	73798542	73799861	chr18	68209247	68210563	1320	1317	0	29	97.87
chrX	51340855	51342167	chrX	52397088	52398400	1313	1313	0	42	99.54
chr1	142482538	142483844	chr1	112966767	112968075	1307	1309	0	62	98.31
chr1	113406945	113408237	chr1	112888249	112889536	1293	1288	0	70	98.21
chr20	23516575	23517866	chr21	23770611	23771915	1292	1305	0	0	85.47
chrX	77257875	77259166	chrX	79754957	79756244	1292	1288	0	2	99.15
chrX	132676327	132677618	chrX	137272334	137273618	1292	1285	0	1	98.99
chr9	5360131	5361421	chr11	5308435	5309722	1291	1288	0	8	98.68
chr6	30476387	30477665	chr5	30951880	30954861	1279	2982	0	56	88.93
chr8	85236924	85238196	chr7	87370891	87372158	1273	1268	0	24	98.26
chr3	23974135	23975374	chr2	24909988	24911228	1240	1241	0	48	95.94
chr11	61765324	61766547	chr9	62801914	62803137	1224	1224	0	0	88.11
chr2	214473644	214474859	chr13	102674243	102675479	1216	1237	0	30	97.03
chr4	70194502	70195711	chr3	76705455	76706658	1210	1204	0	18	98.34
chr16	27123565	27124770	chr18	27822253	27823457	1206	1205	0	49	97
chr9	31418405	31419607	chr11	31890619	31891827	1203	1209	0	12	98.84
chr7	135739254	135740427	chr6	138451212	138452390	1174	1179	0	38	97.96
chr2	53127408	53128580	chr12	54962838	54964010	1173	1173	0	86	96.42
chr5	134526443	134527612	chr4	140874970	140876141	1170	1172	0	10	99.06
chr2	237048672	237049835	chr13	127906469	127907567	1164	1099	0	48	94.9
chrX	133910378	133911529	chrX	138507956	138509091	1152	1136	0	0	90.72
chr2	82061054	82062191	chr12	84887797	84888936	1138	1140	0	10	98.95
chr9	103014613	103015746	chr11	87275547	87276660	1134	1114	0	16	98.64
chr7	5055820	5056938	chr6	5477080	5478198	1119	1119	0	0	98.66
chr18	44758819	44759933	chr17	41043454	41044653	1115	1200	0	27	96.68
chrX	51841203	51842317	chrX	53123250	53124362	1115	1113	0	0	91.7
chr22	22912447	22913559	chr23	21100845	21101996	1113	1152	0	79	93.15
chrX	105328722	105329830	chrX	108893936	108895043	1109	1108	0	0	99.1
chrX	125636238	125637345	chrX	129825662	129826737	1108	1076	0	87	93.8
chr9	87519145	87520242	chr11	71697247	71698390	1098	1144	0	57	91.17
chr2	192911245	192912333	chr13	82807288	82808378	1089	1091	0	40	99.26
chrX	42859196	42860277	chrX	70002508	70003589	1082	1082	0	65	98.8
chrX	50643284	50644353	chrX	51804621	51805697	1070	1077	0	79	88.08
chr1	23014231	23015295	chr1	23370907	23371971	1065	1065	0	6	98.59
chr2	175410035	175411087	chr13	41945350	41946403	1053	1054	0	5	100
chr9	107780931	107781972	chr11	92305187	92306229	1042	1043	0	58	98.75
chrX	148313005	148314039	chrX	153475678	153490667	1035	14990	0	0	97.42

chr4	175268525	175269557	chr3	193246142	193247187	1033	1046	0	53	98.55
chr5	180456457	180457482	chr4	187984873	187985889	1026	1017	0	14	98.62
chr6	168911261	168912284	chr5	173379136	173380147	1024	1012	0	63	98.22
chr14	94477163	94478182	chr15	94914132	94915213	1020	1082	0	55	98.81
chr19	20680643	20681661	chr20	21468782	21469808	1019	1027	0	81	97.84
chr19	39760015	39761030	chr20	36419531	36420522	1016	992	0	0	84.8
chr4	70158092	70159099	chr3	76727244	76728260	1008	1017	0	55	98.61
chr20	1598295	1599301	chr21	1845811	1846807	1007	997	0	8	87.14
chr7	148523227	148524222	chr6	151385191	151386178	996	988	0	85	97.45
chr19	39775373	39776354	chr20	36399224	36399903	982	680	0	25	88.76
chr11	61619092	61620071	chr9	62603599	62604579	980	981	0	69	97.33
chr9	133146111	133147089	chr11	118008080	118009063	979	984	0	61	97.85
chr1	2517646	2518621	chr1	2454344	2455299	976	956	0	43	99.16
chr14	66935373	66936348	chr15	66553854	66554204	976	351	0	20	98.01
chr20	38710454	38711422	chr21	41136679	41137670	969	992	0	16	98.38
chr14	94557546	94558514	chr15	94995463	94996432	969	970	0	77	98.86
chr13	19430565	19431526	chr14	18219844	18220874	962	1031	0	33	87.78
chr8	145115791	145116751	chr7	148393731	148394687	961	957	0	7	98.43
chrX	102275896	102276842	chrX	105868443	105869365	947	923	0	13	88.13
chr11	4492776	4493717	chr9	4615851	4616760	942	910	0	0	82.33
chr21	17267126	17268064	chr22	17345197	17346140	939	944	0	85	94
chr19	21362225	21363162	chr20	22248847	22250431	938	1585	0	0	85.06
chrX	50897515	50898440	chrX	52206357	52207258	926	902	65	77	97.22
chr9	15476003	15476928	chr11	15256631	15256798	926	168	0	6	96.43
chr11	308357	309275	chr9	354244	354986	919	743	0	2	98.65
chr13	64757212	64758128	chr14	47119465	47120381	917	917	0	77	98.91
chr7	143894744	143895647	chr6	146520226	146521133	904	908	0	9	98.67
chr19	15778916	15779816	chr20	16405993	16406893	901	901	0	0	92.45
chr10	65888978	65889873	chr8	66900624	66901508	896	885	0	38	97.4
chr2	242226139	242227030	chr13	133702783	133703688	892	906	0	62	98.21
chr19	49483555	49484446	chr20	46556375	46557179	892	805	0	0	82.21
chr16	72921348	72922230	chr18	63092101	63092982	883	882	0	65	98.97
chr19	20601946	20602824	chr20	21547786	21548666	879	881	0	84	98.75
chr4	117391316	117392194	chr3	134058500	134058973	879	474	0	74	99.58
chr10	133302133	133303009	chr8	136046554	136047425	877	872	0	19	98.39
chr8	55613319	55614194	chr7	57299108	57299992	876	885	0	88	96.8
chr9	34952614	34953488	chr11	35406987	35407874	875	888	0	0	84.9
chr6	31117202	31118074	chr5	31581562	31582432	873	871	0	47	98.74
chr11	61622672	61623543	chr9	62601314	62602182	872	869	0	66	98.85
chr16	73797610	73798480	chr18	68210625	68211494	871	870	0	14	98.74
chrX	148410169	148411021	chrX	153773074	153773922	853	849	0	60	98.82
chrX	149179675	149180515	chrX	154280488	154281328	841	841	0	12	98.93
chr2	130868096	130868934	chr13	17014885	17015726	839	842	0	71	94.07
chr19	47855875	47856711	chr20	44682866	44683699	837	834	0	61	84.36
chr5	168469975	168470808	chr4	175713416	175714249	834	834	0	86	98.92
chr9	137256234	137257064	chr11	122077005	122077828	831	824	0	3	89.79
chrX	123039324	123040153	chrX	127153198	127154021	830	824	0	34	96.24
chr2	174361466	174362294	chr13	63999556	64005200	829	5645	0	76	98.93
chr11	59859223	59860050	chr9	60608080	60608914	828	835	0	29	93.77
chr4	173209827	173210652	chr3	191148500	191149317	826	818	0	37	98.29
chr12	39557826	39558649	chr10	40145243	40146067	824	825	0	37	97.32
chrX	140489952	140490769	chrX	144461907	144462727	818	821	0	36	98.66
chr16	25005168	25005984	chr18	25714020	25714524	817	505	0	47	83.09

chr4	70019362	70020177	chr3	76866451	76867266	816	816	0	0	98.77
chr12	7701754	7702567	chr10	8258688	8259636	814	949	0	46	93.64
chr9	30298832	30299643	chr11	30769125	30769933	812	809	0	75	98.39
chr19	7742484	7743291	chr20	8051718	8052207	808	490	0	60	82.56
chr11	104607594	104608398	chr9	106545199	106545996	805	798	0	25	99.12
chr19	23119367	23120168	chr20	22560799	22561614	802	816	0	0	83.46
chrX	51071273	51072073	chrX	52029082	52029853	801	772	0	0	97.25
chrX	148274716	148275515	chrX	153514651	153515455	800	805	0	0	96.11
chr8	23042799	23043597	chr7	23985543	23986330	799	788	0	0	85.31
chr20	45919641	45920431	chr21	49185704	49186492	791	789	0	0	90.6
chr2	20355597	20356371	chr12	21140865	21141656	775	792	0	84	93.53
chr9	87665459	87666227	chr11	71572419	71577589	769	5171	0	0	82.2
chr11	87850908	87851661	chr9	89685364	89686117	754	754	0	11	98.54
chr1	177547542	177548294	chr1	159917213	159917967	753	755	0	9	98.27
chr2	41106315	41107062	chr12	42653964	42654711	748	748	0	4	97.86
chr3	127330608	127331354	chr2	129131913	129132654	747	742	0	8	89.66
chr9	21420772	21421513	chr11	21592301	21595292	742	2992	0	38	81.8
chr19	39915066	39915806	chr20	36341485	36342252	741	768	0	0	91.6
chr6	107528818	107529558	chr5	109861956	109862620	741	665	0	40	91.91
chrX	56208622	56209356	chrX	57646819	57647547	735	729	0	88	95.2
chr16	26256602	26257334	chr18	26943685	26944418	733	734	0	64	97.68
chr7	6518064	6518796	chr6	6280820	6281545	733	726	0	34	98.62
chr14	105386613	105387344	chr15	106045932	106046668	732	737	0	50	93.7
chr8	39469670	39470400	chr7	40809955	40810677	731	723	0	0	82.24
chr11	4932615	4933344	chr9	4906066	4906795	730	730	0	0	77.5
chr14	28831277	28831999	chr15	27783038	27783761	723	724	0	19	98.06
chr7	88160264	88160978	chr6	89680017	89684121	715	4105	0	20	99.16
chrX	133494739	133495442	chrX	138110598	138110991	704	394	0	43	88.3
chr1	29632006	29632704	chr1	30060652	30068234	699	7583	0	23	85.71
chrX	149053455	149054148	chrX	154409263	154409954	694	692	0	11	98.84
chr1	50850962	50851648	chr1	49056437	49057120	687	684	0	32	99.71
chr22	16133235	16133912	chr23	15992708	15993362	678	655	0	64	98.31
chr19	4581952	4582625	chr20	4859346	4860029	674	684	0	57	97.77
chr19	48561107	48561780	chr20	45468214	45468887	674	674	0	24	98.07
chr19	39762650	39763318	chr20	36484855	36485502	669	648	0	14	89.66
chr3	21507165	21507828	chr2	22380622	22381284	664	663	0	9	99.4
chr16	83746447	83747108	chr18	78663196	78663857	662	662	0	60	98.04
chr6	12540016	12540664	chr5	12826567	12827216	649	650	0	0	99.69
chr20	48820872	48821519	chr21	51424639	51424988	648	350	0	45	94.94
chr2	206896229	206896875	chr13	96974576	96975549	647	974	0	0	87.42
chr2	226241151	226241794	chr13	116825868	116826511	644	644	0	67	99.07
chr19	50319923	50320566	chr20	47462106	47462731	644	626	0	87	93.54
chrX	139228512	139229153	chrX	144064363	144065004	642	642	0	57	99.53
chrX	35628122	35628762	chrX	36913476	36914076	641	601	0	27	78.4
chrX	54740383	54741017	chrX	55952712	55953347	635	636	0	81	90.73
chrX	102258650	102259279	chrX	105707468	105708125	630	658	0	9	81.69
chr7	65058616	65059243	chr6	66708970	66709597	628	628	0	47	91.49
chr11	61722274	61722900	chr9	62796340	62796980	627	641	0	12	83.19
chr21	30719647	30720273	chr22	30712779	30713402	627	624	0	0	84.72
chr6	11122489	11123113	chr5	11396064	11396682	625	619	0	37	98.87
chr6	74035883	74036505	chr5	75163638	75164269	623	632	0	65	95.92
chr19	45681085	45681705	chr20	42639738	42640355	621	618	0	34	98.54
chrX	106281035	106281653	chrX	109849495	109849793	619	299	0	49	88.51

chr20	10736465	10737082	chr21	10614467	10615084	618	618	0	53	97.73
chr6	72090380	72090993	chr5	73222667	73223297	614	631	0	14	96.91
chr11	7755655	7756268	chr9	7874772	7875385	614	614	0	70	98.53
chr11	94180520	94181130	chr9	96105884	96106483	611	600	0	55	98.5
chr10	11532239	11532846	chr8	11672638	11673245	608	608	0	26	98.85
chrX	51839463	51840066	chrX	53125653	53126252	604	600	0	41	89.33
chr12	110828113	110828713	chr10	113454836	113455441	601	606	0	0	99.5
chr3	96465140	96465739	chr2	97543169	97544452	600	1284	0	41	99
chr10	80762191	80762786	chr8	82159759	82160360	596	602	0	78	98.15
chr19	51235299	51235892	chr20	48669814	48670379	594	566	0	0	81.72
chr6	108138043	108138632	chr5	110446950	110447538	590	589	0	40	99.15
chr4	69970449	69971035	chr3	76915217	76915803	587	587	0	0	98.64
chr4	117721769	117722355	chr3	134376569	134377147	587	579	0	14	98.96
chr19	39846637	39847213	chr20	36343053	36343997	577	945	0	2	87.48
chr7	49798764	49799340	chr6	51428289	51428875	577	587	0	82	92.04
chr14	57164584	57165157	chr15	56633946	56634516	574	571	0	64	99.3
chr13	66797282	66797854	chr14	49195163	49195735	573	573	0	50	98.78
chr14	49283221	49283784	chr15	48685252	48685956	564	705	0	53	97.1
chr21	18911765	18912327	chr22	19027124	19027393	563	270	0	0	95.51
chr12	53822381	53822940	chr10	55092819	55093378	560	560	0	6	97.5
chr22	43583047	43583605	chr23	43885379	43885938	559	560	0	65	95.78
chr8	9113380	9113934	chr7	9738879	9739454	555	576	0	64	98.37
chr4	94962348	94962902	chr3	111194246	111194786	555	541	0	27	92.21
chr13	103550518	103551071	chr14	86946211	86946772	554	562	0	0	97.83
chr3	177960921	177961474	chr2	181677532	181678022	554	491	0	56	97.35
chrX	41124130	41124681	chrX	42431748	42432299	552	552	0	58	97.1
chr1	2519272	2519822	chr1	2453143	2453693	551	551	0	79	98.19
chr21	26296030	26296571	chr22	26291208	26291749	542	542	0	0	98.89
chr2	233255894	233256435	chr13	123847301	123847794	542	494	0	65	98.99
chr17	23455023	23455557	chr19	26783649	26784182	535	534	0	27	99.06
chr17	60117316	60117849	chr19	63498832	63499363	534	532	0	55	89.48
chr10	87826478	87827009	chr8	89434962	89435495	532	534	0	63	99.25
chr21	22666856	22667378	chr22	22814875	22815398	523	524	0	80	97.9
chr10	59423942	59424464	chr8	60249392	60249914	523	523	0	5	97.32
chrX	11522793	11523315	chrX	11846105	11846619	523	515	0	33	99.22
chr10	43804716	43805237	chr8	44525849	44526371	522	523	0	18	99.23
chr3	10108076	10108592	chr2	10499107	10499622	517	516	0	37	99.03
chr19	53012031	53012546	chr20	50340265	50340815	516	551	0	48	91.67
chrX	139502798	139503309	chrX	144316574	144317107	512	534	0	53	88.26
chr19	15692471	15692978	chr20	16489892	16491188	508	1297	0	53	84.88
chrX	51913958	51914464	chrX	53050529	53051019	507	491	0	0	92.39
chr21	32196199	32196704	chr22	32173550	32174055	506	506	0	87	97.23
chr2	17431562	17432067	chr12	18253302	18253792	506	491	0	45	83.2
chr11	61768338	61768842	chr9	62799167	62799675	505	509	0	0	84.82
chrX	52506287	52506790	chrX	53626786	53627292	504	507	0	59	87.17
chr13	111949900	111950403	chr14	95583094	95583598	504	505	0	0	98.21
chr3	51446532	51447033	chr2	53043905	53044298	502	394	0	68	88.82
chr13	86076352	86076852	chr14	68819997	68820497	501	501	0	64	99
chr1	105228850	105229346	chr1	104391790	104392264	497	475	0	17	85.61
chr7	4467715	4468210	chr6	4842161	4842640	496	480	0	71	90.89
chr3	32329909	32330398	chr2	33322984	33323473	490	490	0	86	98.98
chrX	117129791	117130280	chrX	121101131	121101620	490	490	0	85	98.57
chr2	17950489	17950977	chr12	18777440	18777923	489	484	0	38	99.17

chr5	2471344	2471829	chr4	2568436	2568921	486	486	0	0	95.27
chr4	136313622	136314106	chr3	153357216	153357699	485	484	0	0	97.52
chr5	180034534	180035015	chr4	185708736	185709408	482	673	0	0	84.9
chr8	118990567	118991046	chr7	121716096	121716577	480	482	0	0	98.54
chr19	51455927	51456405	chr20	48699721	48699820	479	100	0	0	85.37
chrX	50620825	50621298	chrX	51833049	51833703	474	655	0	41	92.49
chr18	18086435	18086903	chr17	13990096	13990564	469	469	0	27	98.29
chr4	89526457	89526925	chr3	105522608	105523073	469	466	0	64	91.61
chr2	225865098	225865565	chr13	116448276	116448743	468	468	0	72	98.93
chr18	53050326	53050792	chr17	49921160	49921626	467	467	0	0	94.22
chrX	54683137	54683600	chrX	56012167	56012636	464	470	0	0	91.3
chr9	102770090	102770551	chr11	87022035	87022496	462	462	0	13	97.84
chr1	214386558	214387018	chr1	198113937	198121952	461	8016	0	63	93.04
chrX	10703036	10703496	chrX	11017130	11017590	461	461	0	0	94.36
chr8	95651898	95652357	chr7	97891418	97891848	460	431	0	66	87.15
chr9	114294256	114294714	chr11	98836036	98836494	459	459	0	25	98.26
chr10	28304028	28304486	chr8	27877016	27877473	459	458	0	75	90.56
chr6	155416581	155417036	chr5	158979949	158980415	456	467	0	35	97.81
chr7	72628485	72628940	chr6	74428787	74429242	456	456	0	0	87.8
chr5	169108397	169108852	chr4	176360756	176361210	456	455	0	32	92.97
chr7	128149123	128149575	chr6	130564926	130565383	453	458	0	14	89.94
chr5	175722812	175723261	chr4	183175597	183176046	450	450	0	10	98.67
chr13	87741132	87741576	chr14	70517141	70517587	445	447	0	0	98.2
chr17	32324600	32325041	chr19	35500777	35501218	442	442	0	26	98.42
chr7	8755426	8755865	chr6	9179645	9180084	440	440	0	51	89.2
chrX	135085218	135085656	chrX	139735942	139736382	439	441	0	0	83.98
chr1	4303571	4304001	chr1	3949111	3949544	431	434	0	0	95.8
chr19	61033519	61033948	chr20	58472963	58473720	430	758	0	0	86.96
chrX	93391342	93391771	chrX	96312741	96313173	430	433	0	74	96.25
chr1	195700589	195701015	chr1	178378515	178378793	427	279	0	31	90.25
chr11	5308631	5309055	chr9	5408231	5408652	425	422	0	0	76.61
chr22	33783556	33783975	chr23	33931439	33931858	420	420	0	71	99.29
chr9	87640955	87641374	chr11	71609655	71610033	420	379	0	0	79.51
chr1	243612883	243613301	chr1	227746552	227746971	419	420	0	49	98.33
chr5	110822860	110823277	chr4	116931655	116932075	418	421	0	35	94.96
chrX	50264659	50265074	chrX	51369475	51369890	416	416	0	40	99.28
chr17	69643821	69644236	chr19	72974571	72974985	416	415	0	0	98.8
chrX	152312263	152312677	chrX	157704203	157704621	415	419	0	0	88.01
chr4	65136859	65137272	chr3	81504583	81504996	414	414	0	26	98.79
chr14	48784628	48785040	chr15	48171749	48172162	413	414	0	46	98.06
chr2	206036015	206036425	chr13	96105630	96106037	411	408	0	74	92.86
chr4	73403961	73404368	chr3	60331225	60331632	408	408	0	89	96.81
chr5	141555451	141555858	chr4	148199108	148199197	408	90	0	75	84.72
chr19	14715164	14715570	chr20	15335312	15335722	407	411	0	0	81.22
chrX	24121089	24121490	chrX	24990287	24990689	402	403	0	19	89.55
chr19	44948129	44948530	chr20	41875857	41876252	402	396	0	0	89.37
chrX	35378813	35379213	chrX	36730859	36731261	401	403	0	26	99
chr19	578051	578451	chr20	627756	628153	401	398	0	75	94.97
chr1	91833917	91834316	chr1	90780893	90781292	400	400	0	78	99.25
chr3	33765539	33765937	chr2	34777932	34778330	399	399	0	58	97.49
chr11	61817080	61817477	chr9	62752963	62753665	398	703	0	28	79.04
chr14	23880644	23881040	chr15	22784974	22785371	397	398	0	0	99.24
chr9	5187454	5187849	chr11	5156659	5157045	396	387	0	0	78.67

chr14	62279662	62280056	chr15	61847509	61847902	395	394	0	38	94.92
chr4	110716391	110716781	chr3	127214619	127215010	391	392	0	74	85.27
chr16	12556861	12557248	chr18	12952461	12952844	388	384	0	0	94.75
chr21	44712843	44713230	chr22	44886454	44886837	388	384	0	15	96.09
chr2	71934236	71934623	chr12	74332659	74333038	388	380	0	0	87.34
chrX	3637642	3638028	chrX	3421113	3421510	387	398	0	43	86.54
chr9	23990900	23991285	chr11	24434474	24434868	386	395	0	13	82.04
chr2	216767738	216768122	chr13	106945213	106945617	385	405	0	79	93.44
chr4	166207360	166207743	chr3	183658772	183659187	384	416	0	0	78.72
chrX	108415444	108415826	chrX	112109130	112109406	383	277	0	83	93.73
chr2	184792030	184792411	chr13	74599115	74599496	382	382	0	0	99.21
chr9	28295841	28296221	chr11	28782288	28782668	381	381	0	22	98.43
chrX	15700103	15700483	chrX	16168208	16168587	381	380	0	72	96.58
chrX	83772163	83772541	chrX	86395427	86395805	379	379	0	0	98.42
chr14	91601653	91602031	chr15	91978955	91979022	379	68	0	76	98.51
chr13	113840040	113840416	chr14	97490865	97491241	377	377	0	0	96.55
chrX	67633776	67634150	chrX	69723559	69723933	375	375	0	0	95.2
chr12	68040855	68041227	chr10	69582248	69582621	373	374	0	70	97.32
chr14	75803110	75803481	chr15	75852161	75852530	372	370	0	82	90.27
chr6	72044609	72044978	chr5	73168791	73169167	370	377	0	26	92.7
chr18	54574647	54575016	chr17	51467753	51468122	370	370	0	70	98.38
chr7	103082342	103082708	chr6	104921604	104921964	367	361	0	0	89.8
chr14	88053915	88054280	chr15	88305132	88305512	366	381	0	28	96.68
chr8	115362180	115362543	chr7	117926286	117926651	364	366	0	0	99.45
chr1	29879461	29879822	chr1	30479398	30479768	362	371	0	30	87.61
chr22	43620922	43621282	chr23	43931098	43931458	361	361	0	0	98.61
chr19	9064848	9065207	chr20	9448254	9448613	360	360	0	0	95.82
chr10	32757660	32758018	chr8	33139698	33140057	359	360	0	45	99.44
chr8	138210629	138210986	chr7	141203488	141203846	358	359	0	22	98.32
chr19	46773741	46774098	chr20	43706266	43706622	358	357	0	0	86.67
chr2	111152313	111152668	chr12	114164407	114164763	356	357	0	0	97.75
chr18	66427200	66427555	chr17	63406534	63406889	356	356	0	1	99.16
chr4	112163159	112163514	chr3	128723608	128723963	356	356	0	0	99.72
chr21	30892802	30893156	chr22	30852081	30852532	355	452	0	0	89.01
chr8	118990212	118990566	chr7	121704776	121705131	355	356	0	85	99.44
chr1	76335600	76335953	chr1	74893099	74893452	354	354	0	34	99.15
chr16	65865089	65865442	chr18	60125845	60126189	354	345	0	79	98.55
chr19	46818123	46818475	chr20	43882940	43883294	353	355	0	0	87.18
chr7	66575949	66576295	chr6	68380697	68381046	347	350	0	8	98.85
chr7	62995497	62995843	chr6	66542426	66542770	347	345	0	78	88.5
chr3	35030469	35030814	chr2	36047376	36047729	346	354	0	7	95.28
chr7	103081995	103082339	chr6	104920778	104921121	345	344	0	0	95.04
chr9	11810114	11810456	chr11	11920466	11920805	343	340	0	47	96.76
chr4	167859368	167859710	chr3	185305521	185305838	343	318	0	65	95.91
chr1	115553438	115553779	chr1	115357991	115358330	342	340	0	0	87.02
chr19	46790422	46790762	chr20	43767792	43768106	341	315	0	1	82.41
chr3	82286240	82286579	chr2	84522462	84524553	340	2092	0	0	99.1
chr11	79625785	79626124	chr9	80980892	80981232	340	341	0	42	93.24
chr8	80823503	80823840	chr7	82952573	82952910	338	338	0	0	98.82
chr10	133303072	133303405	chr8	136046158	136046491	334	334	0	0	97.31
chr8	83725883	83726215	chr7	85802678	85803012	333	335	0	63	98.5
chr2	194553713	194554042	chr13	84466177	84466507	330	331	0	0	95.15
chr1	154855515	154855844	chr1	136906580	136906908	330	329	0	0	99.39

chr2	132016651	132016977	chr13	17917805	17918131	327	327	0	43	98.78
chr6	41284315	41284640	chr5	41889465	41889802	326	338	0	0	93.25
chrX	140491772	140492097	chrX	144460220	144460544	326	325	0	88	97.53
chr13	82171545	82171869	chr14	64879482	64879789	325	308	0	73	98.01
chr4	34623470	34623793	chr3	34936749	34937070	324	322	0	0	93.17
chr9	121640652	121640975	chr11	106377985	106378301	324	317	0	0	96.84
chr8	112883782	112884103	chr7	115388735	115389056	322	322	0	12	95.96
chr9	14058203	14058523	chr11	14260446	14261402	321	957	0	0	90.29
chr2	156445101	156445419	chr13	45633794	45634112	319	319	0	22	98.12
chrY	14306584	14306902	chrY	14967063	14967381	319	319	0	36	95.92
chr6	37638857	37639174	chr5	38126516	38126834	318	319	0	0	97.79
chrX	104626990	104627306	chrX	108087701	108088057	317	357	0	0	83.92
chr11	113877739	113878055	chr9	115882091	115882407	317	317	0	45	97.79
chr1	20594574	20594889	chr1	20910092	20910397	316	306	0	78	97.06
chr6	94773751	94774064	chr5	96520973	96521290	314	318	0	0	99.36
chr2	182572794	182573107	chr13	72321111	72321419	314	309	0	0	89.26
chr7	35266178	35266490	chr6	36438161	36438477	313	317	0	39	100
chr3	185363194	185363506	chr2	189312808	189313120	313	313	0	21	99.36
chr14	41577768	41578078	chr15	40746333	40746643	311	311	0	0	98.71
chr19	59893961	59894271	chr20	57351935	57352242	311	308	0	63	91.61
chr8	9150532	9150840	chr7	9781989	9782300	309	312	0	0	99.35
chr8	85990736	85991044	chr7	88128531	88128839	309	309	0	0	99.03
chr21	44818242	44818550	chr22	44999678	44999959	309	282	0	0	91.76
chr19	57792525	57792832	chr20	55288177	55299083	308	10907	0	20	86.34
chr13	29464463	29464770	chr14	28664262	28664488	308	227	0	60	94.69
chr12	73652434	73652740	chr10	75376587	75376893	307	307	0	41	98.7
chr4	70001224	70001530	chr3	76885193	76885499	307	307	0	83	98.37
chr5	2280237	2280542	chr4	2383967	2384272	306	306	0	61	99.02
chrX	25227544	25227849	chrX	26049182	26049487	306	306	0	0	99.35
chrX	129339967	129340271	chrX	133590000	133590286	305	287	0	0	88.97
chr4	29804041	29804344	chr3	30391196	30391504	304	309	0	0	98.03
chr12	128909455	128909758	chr10	132047397	132047700	304	304	0	0	86
chr4	37219989	37220291	chr3	39885730	39886032	303	303	0	16	97.69
chr1	70579037	70579338	chr1	69238635	69238932	302	298	0	0	83.92
chr6	141661682	141661982	chr5	144559700	144559999	301	300	0	0	98.67
chr6	135072671	135072970	chr5	137848279	137849483	300	1205	0	33	99.31
chr2	18868293	18868592	chr12	19702641	19702940	300	300	0	53	100
chr8	134259439	134259737	chr7	137240092	137240390	299	299	0	0	98.33
chr6	145169404	145169702	chr5	148090480	148090773	299	294	0	0	99.32
chr10	124690604	124690900	chr8	126947929	126948204	297	276	0	0	82.66
chr12	103050593	103050888	chr10	105527281	105527578	296	298	0	17	92.91
chr18	35157793	35158087	chr17	31347646	31347881	295	236	0	58	96.96
chr20	61680512	61680805	chr21	64727448	64727741	294	294	0	0	92.52
chr1	73693631	73693921	chr1	72262542	72262833	291	292	0	0	97.25
chr4	121719951	121720240	chr3	138338342	138338631	290	290	0	0	98.97
chr2	186317954	186318243	chr13	76131413	76131701	290	289	0	0	89.2
chr9	113317360	113317648	chr11	97854577	97854853	289	277	0	62	93.77
chr17	64461759	64462047	chr19	67763523	67763614	289	92	0	63	91.01
chr9	21425344	21425631	chr11	21648765	21649051	288	287	0	68	93.03
chrX	141516183	141516468	chrX	146585710	146585937	286	228	0	32	79.11
chr4	35149794	35150077	chr3	37802922	37803204	284	283	0	0	97.17
chrX	51826820	51827102	chrX	52934670	52934952	283	283	0	0	81.2
chrX	3244862	3245144	chrX	3010641	3010921	283	281	0	0	86.57

chr12	4562669	4562949	chr10	4827563	4827842	281	280	0	0	93.93
chr14	69444425	69444703	chr15	69119854	69120132	279	279	0	44	98.92
chr3	137104791	137105066	chr2	139369425	139369700	276	276	0	0	96.38
chr5	125750968	125751243	chr4	30007891	30008166	276	276	0	14	98.91
chrX	42564213	42564487	chrX	43956731	43957058	275	328	0	1	97.74
chr14	83504719	83504993	chr15	83635449	83635719	275	271	0	26	98.15
chr4	42664715	42664988	chr3	45358275	45358544	274	270	0	52	91.79
chrX	101922666	101922938	chrX	105402879	105403150	273	272	0	67	94.4
chr2	214349388	214349659	chr13	104481406	104481677	272	272	0	0	98.16
chr6	149280432	149280703	chr5	152729677	152729948	272	272	0	4	95.59
chr7	143895670	143895940	chr6	146519934	146520203	271	270	0	0	98.89
chr10	122747720	122747990	chr8	125097294	125097555	271	262	0	0	86.43
chr12	53964237	53964506	chr10	55234572	55234842	270	271	0	0	81.11
chr8	90893025	90893294	chr7	93113774	93114043	270	270	0	85	99.26
chrY	8273131	8273400	chrY	7846316	7846527	270	212	0	38	89.47
chr19	47849556	47849823	chr20	44687429	44687697	268	269	0	4	86.15
chr8	143802241	143802507	chr7	147213786	147214317	267	532	0	0	97.74
chr14	54399273	54399539	chr15	53851740	53852007	267	268	0	83	94.01
chr15	31987740	31988006	chr16	31626198	31626464	267	267	0	0	98.88
chr7	95119714	95119980	chr6	96726824	96727090	267	267	0	0	99.63
chr6	27890665	27890930	chr5	28374211	28374476	266	266	0	0	87.45
chr11	117624560	117624825	chr9	119640221	119640476	266	256	0	0	95.67
chr19	20493206	20493470	chr20	21409382	21409645	265	264	0	39	83.2
chr8	54573512	54573776	chr7	56244720	56244983	265	264	0	40	97.35
chr11	97224350	97224613	chr9	99169731	99169994	264	264	0	0	99.24
chr8	114986483	114986745	chr7	117542152	117542418	263	267	0	40	96.58
chr3	22621122	22621384	chr2	23515948	23516210	263	263	0	30	100
chr11	59787862	59788124	chr9	60779786	60780047	263	262	0	0	86.26
chr21	41815203	41815464	chr22	41850815	41851367	262	553	0	0	87.35
chr19	60566746	60567006	chr20	57992703	57993052	261	350	0	71	90.42
chr5	115158444	115158704	chr4	121328308	121328568	261	261	0	1	98.85
chr7	5808986	5809246	chr6	6993874	6994134	261	261	0	39	97.7
chr1	153083184	153083443	chr1	135044589	135044857	260	269	0	33	98.05
chr3	63196095	63196354	chr2	64961439	64961703	260	265	0	50	91.12
chr1	77545666	77545925	chr1	76114864	76115123	260	260	0	1	98.85
chr3	29483368	29483627	chr2	30418983	30419242	260	260	0	0	96.15
chrX	67691907	67692166	chrX	69664636	69664895	260	260	0	0	97.69
chr4	166260564	166260822	chr3	183650173	183650432	259	260	0	33	89.58
chr9	22325048	22325306	chr11	22594206	22594464	259	259	0	0	98.46
chr11	93314319	93314577	chr9	95198085	95198341	259	257	0	27	93.77
chr2	224663742	224664000	chr13	115244566	115244808	259	243	0	45	91.77
chr13	89063543	89063800	chr14	72000874	72001149	258	276	0	7	90.08
chr3	112062109	112062366	chr2	113617151	113617409	258	259	0	30	86.43
chr4	187377194	187377450	chr3	205596591	205596847	257	257	0	0	98.44
chr4	8574174	8574428	chr3	8649657	8649918	255	262	0	0	89.41
chrX	118919827	118920079	chrX	122940715	122941288	253	574	0	0	85.38
chr11	2155615	2155867	chr9	2352405	2352657	253	253	0	0	98.42
chr15	57825126	57825378	chr16	58192308	58192541	253	234	0	74	89.32
chr16	31520760	31521011	chr18	32562142	32562394	252	253	0	0	95.24
chr2	205463633	205463884	chr13	95475976	95476227	252	252	0	0	98.41
chr7	117863089	117863339	chr6	120180553	120180803	251	251	0	26	98.39
chr3	133246592	133246842	chr2	135343016	135343260	251	245	0	0	90.98
chr8	9197457	9197706	chr7	9828847	9829105	250	259	0	0	85.54

chr10	126829346	126829595	chr8	129341975	129342224	250	250	0	0	98
chr17	64295420	64295669	chr19	67596346	67596595	250	250	0	0	89.02
chr4	33169967	33170216	chr3	33563861	33564110	250	250	0	14	99.2
chrY	8686669	8686918	chrY	8341734	8341978	250	245	0	0	84.9
chr2	213672625	213672873	chr13	103795427	103795741	249	315	0	29	90.61
chr10	96228342	96228590	chr8	97986686	97986934	249	249	0	2	99.2
chr9	74127695	74127943	chr11	57958754	57959002	249	249	0	65	97.59
chr6	161156373	161156621	chr5	164795771	164796016	249	246	0	68	89.14
chrX	113003180	113003428	chrX	116853434	116853679	249	246	0	0	95.12
chr8	97853067	97853315	chr7	100106367	100106611	249	245	0	12	96.73
chr19	2621725	2621972	chr20	2769702	2769916	248	215	0	46	89.11
chr4	110986047	110986294	chr3	127482877	127483065	248	189	0	59	92.02
chr1	43610420	43610666	chr1	44587368	44587624	247	257	0	58	95.95
chr9	101684793	101685039	chr11	85942590	85942836	247	247	0	0	98.79
chr22	34532842	34533087	chr23	34657429	34657673	246	245	0	0	98.78
chr5	144542498	144542741	chr4	151219871	151220117	244	247	0	30	87.97
chr14	22242973	22243216	chr15	21168974	21169217	244	244	0	0	98.36
chr14	67673916	67674158	chr15	67298025	67298267	243	243	0	60	100
chr7	143897623	143897865	chr6	146518101	146518343	243	243	0	66	98.35
chr14	40132921	40133163	chr15	39450970	39451211	243	242	0	68	88.75
chr9	127536467	127536707	chr11	112352524	112352881	241	358	0	0	98.76
chr20	23523955	23524195	chr21	23796151	23796394	241	244	0	0	78.07
chr12	50984367	50984606	chr10	52192037	52192276	240	240	0	0	98.75
chr18	64706235	64706474	chr17	61678306	61678545	240	240	0	0	99.58
chr2	74153649	74153888	chr12	76553524	76553763	240	240	0	0	87.29
chr16	878693	878931	chr18	929272	929508	239	237	0	0	98.31
chr8	143585227	143585465	chr7	146725576	146725812	239	237	0	38	92.8
chr11	58901993	58902230	chr9	59784130	59784400	238	271	0	0	82.61
chr11	25382729	25382966	chr9	25702119	25702356	238	238	0	0	97.9
chr13	111870495	111870732	chr14	95495031	95495268	238	238	0	0	86.13
chr7	54023420	54023657	chr6	55718444	55718681	238	238	0	0	99.16
chrX	12105042	12105279	chrX	12469887	12470124	238	238	0	0	96.64
chr18	41572732	41572968	chr17	37903659	37903903	237	245	0	0	92.83
chr8	90477218	90477454	chr7	92689570	92689804	237	235	0	56	91.91
chr3	95579567	95579801	chr2	96650284	96650546	235	263	0	32	100
chr4	174809222	174809456	chr3	192791874	192792109	235	236	0	0	97.45
chr7	128091513	128091747	chr6	130539567	130539801	235	235	0	0	99.15
chr18	61351670	61351904	chr17	58215144	58215376	235	233	0	0	98.28
chr6	112351077	112351309	chr5	114694666	114694899	233	234	0	31	96.57
chrX	96356407	96356639	chrX	99424610	99424841	233	232	0	0	91.81
chr6	49671643	49671875	chr5	50473624	50473839	233	216	0	59	93.3
chr1	2797390	2797621	chr1	2634006	2634236	232	231	0	0	90
chr8	47728216	47728446	chr7	49220929	49221159	231	231	0	0	93.51
chr1	194489414	194489643	chr1	177143436	177143665	230	230	0	37	98.26
chr16	71915623	71915852	chr18	66289200	66289427	230	228	0	41	86.78
chr5	14861213	14861440	chr4	15344926	15345155	228	230	0	65	93.33
chr19	62183978	62184205	chr20	59920181	59920408	228	228	0	47	96.05
chr19	54596302	54596529	chr20	51954269	51954491	228	223	0	0	85.2
chrX	148291295	148291521	chrX	153512276	153512502	227	227	0	0	96.92
chr15	57717254	57717480	chr16	58135314	58135529	227	216	0	0	99.07
chr10	86972248	86972473	chr8	88572371	88572596	226	226	0	33	96.9
chr9	90007144	90007369	chr11	74185599	74185805	226	207	0	16	92.65
chr11	38414757	38414981	chr9	38896145	38896371	225	227	0	0	92.02

chr20	58799268	58799492	chr21	61799378	61799601	225	224	0	0	87.95
chr7	113714295	113714518	chr6	115768102	115768330	224	229	0	0	90.18
chr22	30840535	30840757	chr23	31012220	31012464	223	245	0	7	84.75
chr8	78185211	78185433	chr7	80259557	80259779	223	223	0	0	88.24
chrX	150360024	150360245	chrX	155626187	155626413	222	227	0	0	88.74
chr9	117726471	117726692	chr11	102325955	102326175	222	221	0	18	98.18
chrX	11172442	11172663	chrX	11515085	11515303	222	219	0	8	81.02
chr8	22861330	22861551	chr7	23802450	23802641	222	192	0	0	87.79
chr4	23330127	23330347	chr3	23851161	23851376	221	216	0	0	92.09
chr19	11695048	11695267	chr20	12161214	12162355	220	1142	0	0	84.65
chr2	50785096	50785315	chr12	52882731	52882950	220	220	0	0	94.55
chr15	66500393	66500611	chr16	67017777	67017995	219	219	0	38	99.09
chr18	50577405	50577623	chr17	47205810	47206028	219	219	0	0	100
chr6	114775806	114776024	chr5	117107397	117107613	219	217	0	0	94.01
chrX	27111187	27111405	chrX	27976209	27976425	219	217	0	26	87.02
chrX	71740012	71740229	chrX	74262961	74263176	218	216	0	59	90.28
chr1	102895295	102895512	chr1	102091664	102091877	218	214	0	12	94.39
chr5	171817898	171818115	chr4	179139711	179139890	218	180	0	0	96.24
chr2	68658078	68658294	chr12	70887032	70887251	217	220	0	0	93.55
chr16	55259676	55259892	chr18	49353514	49353729	217	216	0	0	81.38
chr2	53025566	53025781	chr12	55128563	55128778	216	216	0	0	93.98
chr4	125778829	125779044	chr3	142624574	142624789	216	216	0	0	96.3
chr9	76209279	76209494	chr11	60090241	60090456	216	216	0	13	97.69
chr19	49484447	49484662	chr20	46494324	46494535	216	212	0	0	81.44
chr20	23514528	23514743	chr21	23773543	23773750	216	208	0	0	88.2
chr1	1043879	1044093	chr1	923728	923942	215	215	0	25	96.74
chrX	32869772	32869983	chrX	34069909	34070224	212	316	0	0	87.32
chr18	22425712	22425923	chr17	18355958	18356172	212	215	0	0	87.56
chr5	137046574	137046785	chr4	143647695	143647907	212	213	0	34	97.64
chr16	73527534	73527745	chr18	67917911	67918122	212	212	0	34	97.17
chr8	80183114	80183325	chr7	82306650	82306861	212	212	0	5	87.2
chr2	82523243	82523454	chr12	85356478	85356686	212	209	0	71	96.65
chr17	40573340	40573551	chr19	43853135	43853341	212	207	0	72	98.96
chr9	136831378	136831588	chr11	121608260	121608471	211	212	0	0	97.63
chr2	123047789	123047999	chr13	9069623	9069832	211	210	0	0	91.9
chr20	57961577	57961787	chr21	60965510	60965719	211	210	0	0	88.1
chr3	38137133	38137342	chr2	39448175	39448384	210	210	0	0	92.23
chrX	18882875	18883084	chrX	19611907	19612113	210	207	0	42	92.54
chr1	119519075	119519283	chr1	119364875	119365084	209	210	0	0	98.56
chr14	23012717	23012925	chr15	21979726	21979933	209	208	0	11	99.04
chr2	170783899	170784106	chr13	60402112	60402318	208	207	0	0	98.07
chr6	2674455	2674661	chr5	2687360	2687904	207	545	0	0	97.58
chr4	107032477	107032683	chr3	123477837	123478047	207	211	0	8	98.07
chr13	106474066	106474272	chr14	89878584	89878790	207	207	0	0	100
chr18	20519208	20519414	chr17	16440557	16440759	207	203	0	0	97.54
chr4	109329014	109329219	chr3	125819902	125820107	206	206	0	0	98.54
chr1	159429147	159429352	chr1	141632659	141632863	206	205	0	0	98.54
chr6	27350008	27350213	chr5	27828622	27828824	206	203	0	0	90.15
chr6	135297560	135297764	chr5	138073891	138074095	205	205	0	40	95.12
chr9	104499052	104499256	chr11	88693804	88694008	205	205	0	0	78.89
chr9	24511515	24511719	chr11	24955503	24955697	205	195	0	0	88.08
chr1	204002158	204002361	chr1	187580798	187581005	204	208	0	40	94.61
chr4	152558542	152558745	chr3	169869214	169869417	204	204	0	0	88.24

chr2	239987855	239988057	chr13	130980189	130980391	203	203	0	0	97.54
chr13	27406494	27406696	chr14	26588681	26588853	203	173	0	78	88.82
chr9	5880422	5880623	chr11	5807448	5807649	202	202	0	31	99.5
chr11	117139136	117139336	chr9	119144444	119144644	201	201	0	70	98.01
chrX	111072172	111072372	chrX	114810582	114810780	201	199	0	35	97.99
chr9	127442310	127442509	chr11	112257325	112257550	200	226	0	64	95.79
chrX	113396608	113396807	chrX	117253346	117253545	200	200	0	0	88
chr16	31267199	31267397	chr18	32066103	32066305	199	203	0	66	93.47
chr12	90305218	90305416	chr10	92472745	92472943	199	199	0	0	98.99
chr9	81130243	81130440	chr11	65126962	65127163	198	202	0	58	92.93
chr12	559397	559594	chr10	622099	622296	198	198	0	0	98.99
chr20	58566618	58566814	chr21	61568851	61569041	197	191	0	0	89.53
chrX	37317621	37317816	chrX	38800407	38800602	196	196	0	0	86.22
chrX	53772452	53772647	chrX	55069525	55069718	196	194	0	89	85.33
chr11	13581614	13581808	chr9	13794430	13794630	195	201	0	0	84.44
chr10	31703660	31703854	chr8	32099594	32099789	195	196	0	0	99.49
chr14	91087767	91087961	chr15	91462073	91462268	195	196	0	0	99.48
chr22	29189066	29189260	chr23	29223302	29223490	195	189	0	0	84.04
chr22	45468976	45469169	chr23	45794016	45794210	194	195	0	21	86.44
chr16	70209174	70209366	chr18	64568494	64568689	193	196	0	54	92.19
chr11	70828808	70829000	chr9	72082192	72082385	193	194	0	0	87.05
chr1	37012553	37012745	chr1	37844740	37844932	193	193	0	0	84.21
chr19	57615811	57616003	chr20	55109114	55109306	193	193	0	20	84.21
chrX	8832171	8832363	chrX	9100187	9100379	193	193	0	23	94.3
chr16	24527020	24527211	chr18	25168494	25168772	192	279	0	9	97.79
chr14	67232434	67232625	chr15	66851656	66851854	192	199	0	0	97.92
chr17	69432253	69432444	chr19	72815263	72815454	192	192	0	50	91.15
chr2	187262621	187262812	chr13	77092907	77093098	192	192	0	0	100
chrX	14705889	14706080	chrX	15161552	15161742	192	191	0	0	89.53
chr2	104808412	104808602	chr12	107780507	107780697	191	191	0	0	94.76
chr2	100614747	100614937	chr12	103504945	103505134	191	190	0	0	96.84
chr1	108143728	108143918	chr1	107261432	107261596	191	165	0	49	89.87
chr14	50209204	50209393	chr15	49624803	49624995	190	193	0	1	97.37
chr10	92855040	92855229	chr8	94572500	94572689	190	190	0	0	95.26
chr11	19427536	19427725	chr9	19656036	19656221	190	186	0	0	93.41
chr4	155836980	155837169	chr3	173201431	173201613	190	183	0	42	91.8
chr11	6485142	6485330	chr9	6628568	6628760	189	193	0	0	99.47
chr4	65136339	65136527	chr3	81505328	81505516	189	189	0	0	98.41
chrX	128056560	128056747	chrX	132281797	132281983	188	187	0	0	100
chr1	69076120	69076306	chr1	67641139	67641325	187	187	0	0	94.65
chr14	76101805	76101991	chr15	76158002	76158188	187	187	0	26	96.79
chr4	126872490	126872675	chr3	143726816	143727001	186	186	0	3	94.62
chr6	14395472	14395657	chr5	14691245	14691430	186	186	0	1	98.39
chr22	22937288	22937472	chr23	21092736	21092921	185	186	0	88	93.51
chr4	166033741	166033925	chr3	183498083	183498267	185	185	0	10	95.32
chr1	215328151	215328334	chr1	199065323	199065506	184	184	0	0	99.45
chr4	97869213	97869396	chr3	114198126	114198309	184	184	0	9	98.37
chr3	180175753	180175935	chr2	183940304	183940486	183	183	0	30	93.99
chr13	86154957	86155138	chr14	68948607	68948802	182	196	0	0	93.41
chr13	18464667	18464848	chr14	17365300	17365482	182	183	0	0	91.06
chr1	78762874	78763055	chr1	77355275	77355456	182	182	0	4	99.45
chr16	29798667	29798848	chr18	30560215	30560396	182	182	0	0	95.6
chr18	65022103	65022284	chr17	61998385	61998566	182	182	0	49	97.14

chr16	1064636	1064817	chr18	1124260	1124440	182	181	0	0	93.92
chr6	30647233	30647413	chr5	31095647	31095827	181	181	0	0	94.48
chr10	98511131	98511310	chr8	100218884	100219516	180	633	0	0	86.05
chr7	110130492	110130671	chr6	112083254	112083433	180	180	0	0	97.78
chr11	69468684	69468862	chr9	70590129	70590307	179	179	0	0	96.65
chr11	72036801	72036979	chr9	73310080	73310258	179	179	0	0	95.53
chr17	21091683	21091861	chr19	21461673	21461851	179	179	0	0	93.85
chr2	176498500	176498678	chr13	66135344	66135522	179	179	0	0	98.88
chrY	20889001	20889179	chrY	21952958	21953132	179	175	0	11	95.43
chr11	19138788	19138965	chr9	19368747	19368927	178	181	0	0	95.51
chr19	53342138	53342315	chr20	50610483	50610660	178	178	0	0	97.19
chr4	105103894	105104070	chr3	121498337	121498521	177	185	0	0	94.35
chr1	66188536	66188712	chr1	64543443	64543621	177	179	0	2	96.61
chr7	52570914	52571090	chr6	54244223	54244400	177	178	0	0	96.05
chr15	60243943	60244119	chr16	60585610	60585786	177	177	0	72	91.48
chr1	244226017	244226193	chr1	228306775	228306950	177	176	0	0	89.94
chr3	80723659	80723835	chr2	82962602	82962773	177	172	0	1	98.84
chrX	6991475	6991650	chrX	6950377	6950552	176	176	0	41	95.45
chrX	121730440	121730615	chrX	125803239	125803412	176	174	0	0	81.61
chr9	5221123	5221298	chr11	5130619	5130786	176	168	0	0	84.31
chr1	40144530	40144704	chr1	41030571	41030745	175	175	0	0	96.57
chrX	128651027	128651201	chrX	132890666	132890839	175	174	0	0	85.45
chr5	12415808	12415982	chr4	12818185	12818351	175	167	0	0	100
chr2	22699740	22699913	chr12	23579851	23580251	174	401	0	0	93.33
chr5	11392398	11392571	chr4	11800424	11800597	174	174	0	0	98.85
chr16	71925762	71925934	chr18	66299355	66299527	173	173	0	0	90.75
chr7	71410484	71410656	chr6	73372438	73372608	173	171	0	0	86.98
chr8	2566660	2566831	chr7	2719294	2719465	172	172	0	5	89.53
chr6	27350378	27350549	chr5	27848232	27848394	172	163	0	9	84.66
chr16	68526148	68526318	chr18	62876992	62877162	171	171	0	0	92.4
chr13	90758959	90759129	chr14	73730660	73730815	171	156	0	42	97.44
chr4	107193024	107193194	chr3	123639535	123639679	171	145	0	54	95.8
chr14	53373199	53373367	chr15	52825966	52826135	169	170	0	11	82.25
chr14	30056259	30056427	chr15	29192933	29193101	169	169	0	19	100
chr2	227577488	227577655	chr13	118178987	118179163	168	177	0	11	87.12
chr11	116514073	116514239	chr9	118583053	118583219	167	167	0	0	97.6
chr7	114791781	114791947	chr6	117072595	117072761	167	167	0	0	88.02
chr15	89002567	89002733	chr16	89948906	89949069	167	164	0	0	92.07
chr1	33514766	33514932	chr1	34251929	34252087	167	159	0	22	93.71
chr3	9915141	9915306	chr2	10300422	10300593	166	172	0	0	91.52
chr5	100129956	100130119	chr4	105911888	105912058	164	171	0	0	97.56
chr1	93938407	93938570	chr1	92922493	92922656	164	164	0	53	90.24
chr4	8221157	8221320	chr3	8295540	8295703	164	164	0	0	85.28
chr7	149013579	149013742	chr6	151845643	151845806	164	164	0	0	98.17
chr3	82286077	82286239	chr2	84522094	84522256	163	163	0	0	99.39
chr7	77343889	77344051	chr6	78442718	78442880	163	163	0	0	93.25
chr11	122113391	122113553	chr9	124153464	124153625	163	162	0	4	98.15
chr7	16547484	16547646	chr6	17423144	17423291	163	148	0	56	89.86
chr9	21423241	21423402	chr11	21589109	21589571	162	463	0	0	87.16
chr20	46616227	46616388	chr21	49225235	49225393	162	159	0	0	92.9
chr13	42541997	42542157	chr14	42232097	42232632	161	536	0	0	90.58
chr12	51297947	51298107	chr10	52523553	52523715	161	163	0	0	86.34
chr6	94783813	94783973	chr5	96535797	96535957	161	161	0	41	94.41

chr8	114666412	114666572	chr7	117187697	117187853	161	157	0	0	83.44
chr12	113433271	113433431	chr10	116153399	116153548	161	150	0	78	86.3
chrX	35240989	35241148	chrX	36590163	36590333	160	171	0	13	94.38
chr1	181689578	181689737	chr1	164258649	164258809	160	161	0	0	83.12
chr1	3712839	3712998	chr1	3566667	3566826	160	160	0	0	89.38
chr6	6467206	6467365	chr5	6832272	6832429	160	158	0	0	98.73
chr14	73314839	73314998	chr15	73098042	73098198	160	157	0	0	89.54
chr21	31631432	31631591	chr22	31599198	31599353	160	156	0	0	90.38
chr8	1705502	1705660	chr7	1785222	1785380	159	159	0	0	99.37
chr10	104491132	104491290	chr8	106245410	106245566	159	157	0	2	94.87
chr2	205463415	205463572	chr13	95483199	95483356	158	158	0	0	94.3
chr2	236060835	236060992	chr13	126785477	126785634	158	158	0	0	93.04
chr8	67568338	67568495	chr7	69577675	69577832	158	158	0	0	100
chr19	53056399	53056555	chr20	50317226	50317430	157	205	0	0	83.44
chr10	29326077	29326233	chr8	29674863	29675030	157	168	0	0	87.26
chrX	150100730	150100886	chrX	155399717	155399874	157	158	0	24	83.44
chr13	66783979	66784135	chr14	49179917	49180073	157	157	0	0	98.73
chr6	86971288	86971444	chr5	88676075	88676231	157	157	0	1	97.45
chr20	32507822	32507978	chr21	34516240	34516392	157	153	0	0	96.03
chr6	167252931	167253086	chr5	171609339	171609495	156	157	0	21	85.9
chr5	119186456	119186611	chr4	125419844	125419999	156	156	0	0	95.51
chr10	7182872	7183027	chr8	7287504	7287658	156	155	0	2	89.03
chrX	3335009	3335164	chrX	3098555	3098706	156	152	0	0	89.47
chr16	27949423	27949577	chr18	28648258	28648412	155	155	0	61	98.06
chr2	77137794	77137948	chr12	79928535	79928689	155	155	0	26	94.84
chr7	117944912	117945066	chr6	120261783	120261937	155	155	0	20	84.52
chr17	24502037	24502190	chr19	27850250	27850408	154	159	0	0	94.16
chr4	256818	256971	chr3	252230	252387	154	158	0	0	83.77
chr11	93688501	93688654	chr9	95589807	95589960	154	154	0	0	98.7
chr20	57347040	57347193	chr21	60366280	60366433	154	154	0	41	100
chr6	78793908	78794061	chr5	80229291	80229444	154	154	0	0	99.35
chr3	125892323	125892475	chr2	127718878	127719030	153	153	0	42	99.35
chr5	168102074	168102226	chr4	175320939	175321091	153	153	0	0	98.69
chr6	97793236	97793387	chr5	99757466	99757937	152	472	0	0	94.67
chr3	128938668	128938819	chr2	130814301	130814452	152	152	0	60	90.79
chrX	81135464	81135615	chrX	83675759	83675896	152	138	0	43	91.11
chr11	25876225	25876375	chr9	26170571	26170723	151	153	0	47	99.34
chr11	4924732	4924882	chr9	4905915	4906065	151	151	0	0	77.48
chr2	57490873	57491023	chr12	59589906	59590054	151	149	0	4	100
chr19	46880401	46880550	chr20	43837614	43838786	150	1173	0	0	83.16
chr19	2495976	2496125	chr20	2711747	2711898	150	152	0	0	94
chr19	2586362	2586511	chr20	2631917	2632064	150	148	0	0	82.43
chr15	83320279	83320427	chr16	84033920	84034067	149	148	0	0	87.84
chr11	33668022	33668169	chr9	34051434	34051586	148	153	0	0	88.36
chr12	91257237	91257384	chr10	93432386	93432537	148	152	0	0	93.92
chr12	76245704	76245851	chr10	77781629	77781776	148	148	0	49	99.32
chr14	47972799	47972946	chr15	47343202	47343348	148	147	0	3	92.52
chrX	130690637	130690783	chrX	134945751	134945915	147	165	0	0	79.45
chr13	105806005	105806151	chr14	89209435	89209581	147	147	0	0	90.48
chr19	57832022	57832168	chr20	55248042	55248188	147	147	0	67	91.84
chr20	5502021	5502167	chr21	5487022	5487168	147	147	0	10	98.64
chr8	876482	876628	chr7	924726	924872	147	147	0	0	97.28
chr16	3797266	3797411	chr18	3661926	3662071	146	146	0	0	98.63

chr18	69215538	69215683	chr17	66378011	66378156	146	146	0	0	94.52
chr9	108884845	108884989	chr11	93417084	93417257	145	174	0	22	88.97
chr11	42337395	42337539	chr9	42811330	42811495	145	166	0	53	99.31
chr19	57833290	57833434	chr20	55246860	55247009	145	150	0	0	84.83
chr11	132372684	132372828	chr9	134543911	134544055	145	145	0	0	97.24
chr19	2044332	2044476	chr20	2154203	2154347	145	145	0	0	95.17
chr2	75227541	75227685	chr12	77633749	77633893	145	145	0	0	96.55
chrX	146078312	146078456	chrX	151257054	151257198	145	145	0	0	97.93
chr8	138801320	138801464	chr7	141783271	141783412	145	142	0	0	97.18
chr1	188522225	188522368	chr1	171088101	171088245	144	145	0	0	83.92
chr11	15961188	15961331	chr9	16167560	16167675	144	116	0	0	94.83
chr3	183932800	183932942	chr2	187808958	187809101	143	144	0	0	86.62
chr1	201910290	201910431	chr1	184863443	184863584	142	142	0	0	99.3
chr9	85424726	85424867	chr11	69474995	69475136	142	142	0	0	96.48
chr19	60828088	60828229	chr20	58272207	58272311	142	105	0	47	79.05
chr4	38298611	38298751	chr3	40970658	40970809	141	152	0	0	92.91
chr16	9224259	9224399	chr18	9355026	9355166	141	141	0	0	89.36
chr5	177778778	177778918	chr4	185287494	185287634	141	141	0	0	90
chr6	34286789	34286929	chr5	34737047	34737187	141	141	0	25	99.29
chr9	102982367	102982507	chr11	87245318	87245458	141	141	0	0	90.78
chr9	135034511	135034651	chr11	119873162	119873302	141	141	0	61	97.16
chr13	112329733	112329873	chr14	95940754	95940890	141	137	0	1	91.97
chr22	36384008	36384147	chr23	36559279	36559418	140	140	0	2	93.57
chr11	116512818	116512957	chr9	118584331	118584469	140	139	0	28	98.55
chrX	21581982	21582121	chrX	22345034	22345166	140	133	0	0	89.47
chr2	228414656	228414795	chr13	119014989	119015104	140	116	0	0	94.83
chr20	35920566	35920704	chr21	38291240	38291378	139	139	0	0	95.65
chr19	39763319	39763457	chr20	36417158	36417295	139	138	0	75	92.75
chr7	118306227	118306365	chr6	120308092	120308229	139	138	0	0	78.2
chr2	141339667	141339805	chr13	30368331	30368467	139	137	0	65	94.4
chrX	6947723	6947861	chrX	6909419	6909555	139	137	0	0	74.45
chr3	128578682	128578820	chr2	130455392	130455515	139	124	0	45	95.16
chr1	68832216	68832353	chr1	67395211	67395348	138	138	0	62	95.65
chr13	21216355	21216492	chr14	20379764	20379901	138	138	0	0	94.93
chr11	59863345	59863482	chr9	60600996	60601132	138	137	0	56	91.11
chr2	219049904	219050040	chr13	109205786	109205923	137	138	0	2	89.05
chrX	35624023	35624159	chrX	36919637	36919774	137	138	0	0	82.48
chr4	123352382	123352518	chr3	140188304	140188440	137	137	0	0	96.35
chr2	80573531	80573666	chr12	83389412	83389547	136	136	0	0	97.06
chr6	124730688	124730823	chr5	127255302	127255437	136	136	0	23	99.26
chr4	171630449	171630583	chr3	189298799	189298940	135	142	0	0	94.81
chr10	56440423	56440557	chr8	57371148	57371286	135	139	0	0	99.26
chr1	185708293	185708427	chr1	168279683	168279817	135	135	0	0	93.18
chr3	128429139	128429273	chr2	130258739	130258869	135	131	0	0	87.69
chr8	106889337	106889470	chr7	109325452	109325591	134	140	0	0	85.82
chr1	105341314	105341447	chr1	104277320	104277453	134	134	0	0	83.33
chr13	33053773	33053906	chr14	32256415	32256548	134	134	0	0	92.54
chr14	39937257	39937390	chr15	39277879	39278012	134	134	0	0	99.25
chr16	24253882	24254015	chr18	24891280	24891413	134	134	0	41	92.54
chr7	113375773	113375906	chr6	115417325	115417454	134	130	0	0	98.46
chrX	104382801	104382934	chrX	107842105	107842231	134	127	0	0	88.98
chr2	143564201	143564333	chr13	32607776	32607916	133	141	0	9	92.48
chr14	61574587	61574719	chr15	61143072	61143206	133	135	0	0	87.9

chr1	222385121	222385253	chr1	206209622	206209754	133	133	0	0	90.23
chr2	115513986	115514118	chr13	1527264	1527396	133	133	0	40	96.24
chr6	141661983	141662115	chr5	144560012	144560144	133	133	0	0	98.5
chr9	135149889	135150021	chr11	120039201	120039333	133	133	0	0	95.49
chr1	226462087	226462218	chr1	210309939	210310075	132	137	0	0	93.89
chr6	78371556	78371687	chr5	79800141	79800274	132	134	0	0	93.18
chr10	132476001	132476132	chr8	135189667	135189797	132	131	0	0	96.95
chrX	67033302	67033432	chrX	69070358	69070490	131	133	0	0	93.13
chrX	84152421	84152551	chrX	86796583	86796715	131	133	0	2	95.16
chr15	43697607	43697737	chr16	43645304	43645434	131	131	0	1	96.95
chr16	73978238	73978368	chr18	68376167	68376297	131	131	0	0	91.6
chr2	126834986	126835116	chr13	12928119	12928249	131	131	0	0	98.47
chr9	137260037	137260167	chr11	122074471	122074601	131	131	0	43	97.71
chr20	30573956	30574086	chr21	32272703	32272832	131	130	0	0	91.54
chr10	132828459	132828588	chr8	135556758	135556902	130	145	0	0	91.41
chr3	135058185	135058314	chr2	137212613	137212743	130	131	0	0	96.15
chr19	62976975	62977104	chr20	60647699	60647827	130	129	0	0	80.17
chr4	163381483	163381611	chr3	180828808	180829028	129	221	0	49	92.97
chr15	91519693	91519821	chr16	92495572	92495700	129	129	0	1	90.7
chr6	101214450	101214578	chr5	103194566	103194694	129	129	0	0	93.8
chr3	175096593	175096720	chr2	178678750	178678880	128	131	0	0	85.16
chr11	31049678	31049805	chr9	31383872	31383999	128	128	0	7	91.41
chr2	82511102	82511229	chr12	85343516	85343643	128	128	0	0	97.66
chr2	150970923	150971050	chr13	40025740	40025867	128	128	0	0	100
chr3	124577756	124577883	chr2	126277582	126277709	128	128	0	0	92.19
chrX	43581698	43581825	chrX	45023394	45023521	128	128	0	0	85.94
chrX	37381039	37381165	chrX	38483251	38483378	127	128	0	0	83.46
chr1	207473116	207473242	chr1	191083070	191083196	127	127	0	18	100
chr11	5657116	5657242	chr9	5809564	5809685	127	122	0	0	74.78
chr19	44431275	44431401	chr20	41390860	41390980	127	121	0	0	97.52
chr21	38499164	38499290	chr22	39166942	39167059	127	118	0	0	91.89
chr16	86671116	86671241	chr18	81639607	81639734	126	128	0	0	99.21
chr3	6367679	6367804	chr2	6582977	6583103	126	127	0	0	88.89
chr13	102436178	102436303	chr14	85827368	85827493	126	126	0	0	91.27
chr19	48560172	48560297	chr20	45469700	45469825	126	126	0	19	96.8
chr3	69945219	69945344	chr2	71754695	71754820	126	126	0	0	87.3
chr11	4799873	4799997	chr9	4824382	4824506	125	125	0	0	81.6
chr11	42745732	42745856	chr9	43225896	43226020	125	125	0	0	88.8
chr12	111537987	111538111	chr10	114236614	114236738	125	125	0	0	89.6
chr13	104447844	104447968	chr14	87852231	87852355	125	125	0	0	94.4
chrX	9275329	9275452	chrX	9591576	9591709	124	134	0	0	94.31
chr1	231579103	231579226	chr1	215400109	215400232	124	124	0	0	96.77
chr7	37927227	37927350	chr6	39118836	39118959	124	124	0	21	93.55
chr14	65398909	65399031	chr15	65001649	65001771	123	123	0	0	94.31
chr4	96649338	96649460	chr3	112943838	112943960	123	123	0	0	98.37
chr4	139261019	139261141	chr3	156436842	156436964	123	123	0	0	95.12
chr11	46127235	46127356	chr9	46630624	46630803	122	180	0	0	97.52
chr9	135192451	135192572	chr11	120081561	120081712	122	152	0	0	93.33
chr2	131318123	131318244	chr13	17206751	17206873	122	123	0	0	90.98
chr11	6746121	6746242	chr9	6908108	6908229	122	122	0	0	90.18
chrX	62763846	62763967	chrX	64556207	64556328	122	122	0	0	96.72
chr14	93746419	93746539	chr15	94194568	94194690	121	123	0	0	96.69
chrX	35423827	35423947	chrX	36775983	36776103	121	121	0	6	89.26

chrX	77670811	77670931	chrX	80155082	80155201	121	120	0	0	98.32
chrX	130687614	130687734	chrX	134945919	134946024	121	106	0	0	81.13
chr8	37482513	37482632	chr7	38704730	38704851	120	122	0	0	85.83
chr9	6039070	6039189	chr11	5965070	5965191	120	122	0	0	94.12
chr5	125881312	125881431	chr4	132212606	132212725	120	120	0	70	83.33
chr6	37130125	37130244	chr5	37606978	37607097	120	120	0	0	98.33
chr6	115785592	115785711	chr5	118129682	118129801	120	120	0	18	97.5
chr19	33242344	33242462	chr20	29422463	29422585	119	123	0	0	80.67
chrX	99997016	99997134	chrX	103255440	103255561	119	122	0	0	90.76
chr6	32669576	32669694	chr5	33124719	33124838	119	120	0	0	91.6
chr21	39806178	39806296	chr22	39847879	39847997	119	119	0	0	92.44
chrX	116626112	116626230	chrX	120573263	120573377	119	115	0	0	87.72
chr3	79029433	79029550	chr2	81247582	81247703	118	122	0	37	91.53
chr13	99420703	99420820	chr14	82762397	82762516	118	120	0	3	86.67
chr3	28726355	28726472	chr2	29669228	29669345	118	118	0	86	88.98
chr12	40902521	40902638	chr10	41500275	41500391	118	117	0	0	82.46
chr21	30778603	30778720	chr22	30775031	30775143	118	113	0	5	95.58
chr8	79278447	79278563	chr7	81399492	81399609	117	118	0	0	91.45
chr7	18248427	18248543	chr6	19154385	19154501	117	117	0	78	91.45
chr3	185498313	185498429	chr2	189454777	189454890	117	114	0	0	100
chrX	135812059	135812175	chrX	140465540	140465652	117	113	0	0	86.24
chr2	186192034	186192149	chr13	76006518	76006634	116	117	0	41	90.18
chr3	160103702	160103817	chr2	163284067	163284183	116	117	0	0	97.41
chr6	112375533	112375648	chr5	114721691	114721807	116	117	0	0	89.66
chr19	47777935	47778050	chr20	44767233	44767348	116	116	0	78	87.93
chr2	224061428	224061543	chr13	114653717	114653832	116	116	0	0	93.1
chr2	238835880	238835995	chr13	129779096	129779210	116	115	0	0	87.83
chr7	98965208	98965322	chr6	100860118	100860236	115	119	0	0	80.95
chr2	129115899	129116013	chr13	15249309	15249424	115	116	0	7	89.57
chr1	225615996	225616110	chr1	209437120	209437234	115	115	0	0	97.39
chrX	83971853	83971967	chrX	86595936	86596050	115	115	0	0	92.17
chr7	118296532	118296646	chr6	120317559	120317672	115	114	0	0	84.16
chr19	14853054	14853167	chr20	15538669	15538842	114	174	0	0	80.7
chr12	103325843	103325956	chr10	105803185	105803298	114	114	0	0	100
chr2	145462898	145463011	chr13	34500153	34500266	114	114	0	0	96.49
chr6	107227357	107227470	chr5	109387644	109387757	114	114	0	0	92.92
chr5	178627320	178627432	chr4	186133362	186134338	113	977	0	0	91.84
chr4	153390959	153391071	chr3	170710291	170710435	113	145	0	47	87.5
chr2	163313008	163313120	chr13	52712642	52712754	113	113	0	0	92.92
chr4	164276243	164276355	chr3	181732394	181732506	113	113	0	1	96.46
chr10	107217518	107217630	chr8	109019523	109019634	113	112	0	28	100
chr15	50973769	50973881	chr16	51050113	51050223	113	111	0	0	93.69
chr1	196312313	196312425	chr1	178999697	178999804	113	108	0	41	98.15
chr12	123537412	123537523	chr10	126609309	126609423	112	115	0	0	93.75
chr2	204779501	204779612	chr13	94780839	94780952	112	114	0	0	86.61
chr8	34828264	34828375	chr7	36023493	36023604	112	112	0	0	98.21
chr9	122515337	122515448	chr11	107228985	107229096	112	112	0	48	97.32
chr9	74437243	74437354	chr11	58267882	58267991	112	110	0	75	93.64
chr5	168938136	168938246	chr4	176201168	176201278	111	111	0	59	100
chr7	117862978	117863088	chr6	120168107	120168217	111	111	0	0	97.3
chr3	22119406	22119515	chr2	22997300	22997414	110	115	0	22	92.73
chr4	93927356	93927465	chr3	110044882	110044993	110	112	0	26	97.27
chr8	20399945	20400054	chr7	21291406	21291516	110	111	0	0	94.55

chr11	54864808	54864917	chr9	55443672	55443781	110	110	0	0	97.27
chr16	23056295	23056404	chr18	23670454	23670563	110	110	0	13	94.55
chr17	69228047	69228156	chr19	72605522	72605631	110	110	0	0	81.82
chr2	22562300	22562409	chr12	23439726	23439835	110	110	0	0	99.09
chr6	25839990	25840099	chr5	26784644	26784753	110	110	0	0	91.82
chrX	129585723	129585832	chrX	133826783	133826892	110	110	0	0	94.55
chr20	55092872	55092981	chr21	58016696	58016804	110	109	0	0	91.74
chr8	141205135	141205244	chr7	144244527	144244635	110	109	0	0	98.17
chr11	42223858	42223967	chr9	42696827	42696933	110	107	0	0	90.65
chr18	60865429	60865538	chr17	57728039	57728136	110	98	0	4	84.69
chr1	178332038	178332146	chr1	160714405	160714518	109	114	0	0	95.41
chr6	20683675	20683783	chr5	21080557	21080665	109	109	0	0	93.52
chrX	127268928	127269036	chrX	131482764	131482865	109	102	0	0	89.22
chr10	10434857	10434964	chr8	10557455	10557564	108	110	0	0	91.67
chr2	222968441	222968548	chr13	113535682	113535789	108	108	0	0	94.44
chr10	65728411	65728518	chr8	66738566	66738672	108	107	0	0	83.96
chr20	55852487	55852594	chr21	58781959	58782064	108	106	0	0	92.45
chr3	20362741	20362847	chr2	21230311	21230421	107	111	0	18	100
chr14	58220612	58220718	chr15	57791272	57791378	107	107	0	10	99.07
chr19	46880294	46880400	chr20	43793394	43793500	107	107	0	0	85.42
chr4	175736380	175736486	chr3	193722952	193723058	107	107	0	0	94.39
chr4	86821791	86821896	chr3	102823800	102823905	106	106	0	0	83.81
chr9	79669695	79669800	chr11	63576262	63576367	106	106	0	0	97.17
chrX	43390673	43390778	chrX	44794317	44794422	106	106	0	0	93.4
chr8	127715925	127716030	chr7	130427497	130427601	106	105	0	1	93.33
chr19	49484663	49484768	chr20	46594354	46594453	106	100	0	0	76.04
chr2	18693050	18693154	chr12	19527822	19527930	105	109	0	0	92.38
chr19	14800097	14800201	chr20	15538506	15538612	105	107	0	0	72.38
chr14	23114770	23114874	chr15	22082567	22082671	105	105	0	0	87.62
chr3	13108993	13109097	chr2	13680855	13680959	105	105	0	8	92.38
chr5	15501717	15501821	chr4	15921168	15921272	105	105	0	10	98.1
chr15	79465018	79465121	chr16	80131058	80131161	104	104	0	0	91.35
chr6	8929968	8930071	chr5	9301907	9302010	104	104	0	0	100
chr10	111249046	111249148	chr8	113074403	113074507	103	105	0	0	84.16
chr1	98592787	98592889	chr1	97624766	97624868	103	103	0	0	98.06
chr11	85523229	85523331	chr9	87170029	87170131	103	103	0	0	99.03
chr2	13910316	13910417	chr12	14681157	14681258	102	102	0	2	97.06
chr19	57521015	57521115	chr20	55114274	55114379	101	106	0	0	80.2
chr14	91087962	91088062	chr15	91462555	91462655	101	101	0	0	98.02
chr3	126165478	126165578	chr2	127995567	127995667	101	101	0	0	94.06
chr11	66167727	66167827	chr9	67441606	67441703	101	98	0	0	84.44
chr12	106613274	106613373	chr10	109127183	109127282	100	100	0	0	99
chr5	2308815	2308914	chr4	2415278	2415377	100	100	0	0	98
chr6	102037372	102037470	chr5	104021672	104022502	99	831	0	0	100
chr19	39903474	39903572	chr20	36350581	36350681	99	101	0	0	91.92
chr2	30237432	30237530	chr12	31239584	31239682	99	99	0	0	89.9
chr2	47326911	47327009	chr12	49359512	49359610	99	99	0	4	96.97
chr4	178028710	178028808	chr3	196012917	196013015	99	99	0	0	92.93
chrX	44474325	44474423	chrX	45934051	45934149	99	99	0	14	96.97
chrX	54697577	54697675	chrX	55993208	55993306	99	99	0	0	88.89
chr19	22408054	22408151	chr20	23353153	23353303	98	151	0	36	85.71
chr21	38499291	38499388	chr22	39189416	39189516	98	101	0	0	90.82
chr1	202402588	202402685	chr1	185361977	185362074	98	98	0	0	92.86

chr11	68839128	68839225	chr9	69906837	69906934	98	98	0	0	86.73
chr2	28058796	28058893	chr12	29005853	29005950	98	98	0	0	84.69
chr21	22751270	22751367	chr22	22901255	22901352	98	98	0	76	97.96
chr4	160028945	160029042	chr3	177457952	177458049	98	98	0	36	92.86
chr6	35265020	35265117	chr5	35722724	35722821	98	98	0	0	97.96
chr16	3170240	3170337	chr18	3054237	3054333	98	97	0	0	84.78
chr15	67417445	67417541	chr16	68013754	68013852	97	99	0	76	93.68
chr10	658392	658488	chr8	718955	719051	97	97	0	0	86.6
chr10	103885725	103885821	chr8	105644500	105644596	97	97	0	0	91.75
chr22	21795003	21795099	chr23	21900216	21900312	97	97	0	0	90.72
chr6	104244023	104244119	chr5	106264349	106264445	97	97	0	0	94.85
chrX	102022820	102022916	chrX	105669108	105669204	97	97	0	0	83.51
chr10	32091405	32091500	chr8	32472103	32472201	96	99	0	0	90.43
chr1	91913840	91913935	chr1	90870499	90870594	96	96	0	0	100
chr2	134805569	134805664	chr13	23802065	23802160	96	96	0	0	100
chr4	95237011	95237106	chr3	111451872	111451967	96	96	0	6	98.96
chr1	14322076	14322171	chr1	14378857	14378951	96	95	0	0	94.74
chr16	71695493	71695588	chr18	66060757	66060851	96	95	0	0	89.47
chr2	13583930	13584025	chr12	14355073	14355165	96	93	0	3	94.62
chr2	231633341	231633436	chr13	122252296	122252388	96	93	0	7	79.76
chr20	32590022	32590116	chr21	34600546	34600640	95	95	0	0	94.74
chr4	130966020	130966114	chr3	147908586	147908680	95	95	0	0	94.74
chr15	92433828	92433922	chr16	93404793	93404884	95	92	0	0	92.39
chrX	20191515	20191608	chrX	20928012	20928106	94	95	0	0	90
chr1	158185596	158185689	chr1	140233414	140233507	94	94	0	0	94.68
chr11	71949788	71949881	chr9	73223438	73223531	94	94	0	0	84.04
chr11	75711048	75711141	chr9	77005306	77005399	94	94	0	0	92.55
chr12	128374673	128374766	chr10	131505504	131505597	94	94	0	0	97.87
chr4	116259406	116259499	chr3	132938375	132938468	94	94	0	24	96.81
chrX	129606896	129606989	chrX	133850847	133850940	94	94	0	0	89.25
chr21	27062957	27063050	chr22	27057528	27057620	94	93	0	4	91.4
chr7	66713069	66713162	chr6	68520704	68520795	94	92	0	0	90.22
chr16	57710665	57710757	chr18	51844661	51844759	93	99	0	0	86.96
chr11	23002516	23002608	chr9	23276856	23276949	93	94	0	0	90.32
chr11	23925752	23925844	chr9	24260031	24260123	93	93	0	0	100
chr3	69046952	69047044	chr2	70832958	70833050	93	93	0	0	93.55
chr7	135115051	135115143	chr6	137818166	137818258	93	93	0	0	96.77
chr10	122437223	122437314	chr8	124755763	124755878	92	116	0	0	96.74
chr2	212693857	212693948	chr13	102797011	102797102	92	92	0	28	100
chr3	178582302	178582393	chr2	182312214	182312305	92	92	0	0	94.57
chr6	84072796	84072887	chr5	85709245	85709336	92	92	0	0	100
chr6	140638271	140638362	chr5	143528582	143528673	92	92	0	0	91.3
chr4	134461779	134461869	chr3	151447480	151447566	91	87	0	0	98.84
chr1	156627918	156628007	chr1	138707033	138707125	90	93	0	59	88.89
chr3	96204791	96204880	chr2	97282180	97282269	90	90	0	29	91.11
chr5	1253950	1254039	chr4	1285117	1285206	90	90	0	0	90
chr1	236485202	236485291	chr1	220401014	220401098	90	85	0	0	79.76
chr3	85836559	85836647	chr2	88141592	88141681	89	90	0	0	94.38
chr1	66377420	66377508	chr1	64730442	64730530	89	89	0	0	97.75
chr19	53219281	53219369	chr20	50488376	50488464	89	89	0	0	95.51
chr2	3788096	3788184	chr12	4419214	4419302	89	89	0	87	82.02
chr20	11466691	11466779	chr21	11337666	11337754	89	89	0	68	96.63
chr4	34391508	34391596	chr3	34792995	34793083	89	89	0	0	95.51

chr5	110649343	110649431	chr4	116760867	116760955	89	89	0	0	98.88
chr6	69459242	69459330	chr5	70569697	70569785	89	89	0	0	98.88
chr8	75000285	75000373	chr7	77039920	77040007	89	88	0	0	96.59
chr1	89512494	89512581	chr1	88122074	88122161	88	88	0	0	81.82
chr11	44099227	44099314	chr9	44596915	44597002	88	88	0	0	96.59
chr14	86325651	86325738	chr15	86560887	86560974	88	88	0	0	88.64
chr4	124689928	124690015	chr3	141517088	141517175	88	88	0	0	88.64
chr6	120771774	120771861	chr5	123268975	123269062	88	88	0	0	96.59
chr7	8757211	8757298	chr6	9178195	9178282	88	88	0	0	87.5
chr11	66263456	66263543	chr9	67512683	67512768	88	86	0	0	95.35
chr8	65411772	65411858	chr7	67414353	67414439	87	87	0	0	97.7
chr8	121488270	121488356	chr7	124138043	124138129	87	87	0	0	100
chr1	11642112	11642197	chr1	11856119	11856204	86	86	0	0	86.05
chr10	70890246	70890331	chr8	72045445	72045530	86	86	0	0	88.37
chr2	104212336	104212421	chr12	107145328	107145413	86	86	0	0	93.02
chr8	49190609	49190694	chr7	50753338	50753423	86	86	0	0	95.35
chr2	205309948	205310033	chr13	95323076	95323160	86	85	0	0	97.65
chr3	134331406	134331491	chr2	136473010	136473094	86	85	0	0	97.65
chr6	27756851	27756936	chr5	28239810	28239862	86	53	0	84	92.45
chr8	33960604	33960688	chr7	35144690	35144778	85	89	0	0	96.47
chr12	56543664	56543748	chr10	57789073	57789157	85	85	0	0	88.24
chr13	107708717	107708801	chr14	91116757	91116841	85	85	0	0	89.41
chr14	30056428	30056512	chr15	29203848	29203932	85	85	0	0	97.65
chr4	27044164	27044248	chr3	27618893	27618977	85	85	0	0	96.47
chr4	180711378	180711462	chr3	198746826	198746910	85	85	0	0	89.41
chr13	101588103	101588187	chr14	84938798	84938880	85	83	0	0	97.59
chr3	22738720	22738804	chr2	23649058	23649140	85	83	0	0	86.75
chr15	54303233	54303316	chr16	54650726	54650810	84	85	0	0	98.81
chr1	68441398	68441481	chr1	67002182	67002265	84	84	0	0	95.06
chr9	101721227	101721310	chr11	85980515	85980598	84	84	0	0	96.43
chrX	128574595	128574678	chrX	132808936	132809019	84	84	0	25	88.1
chrX	154463142	154463225	chrX	159778832	159778915	84	84	0	0	100
chr4	88657696	88657778	chr3	104646603	104646688	83	86	0	0	93.98
chr10	117877464	117877546	chr8	120049634	120049716	83	83	0	0	100
chr14	101991229	101991311	chr15	102601191	102601273	83	83	0	0	85.19
chr9	74671611	74671693	chr11	58504362	58504444	83	83	0	0	98.8
chr1	29450857	29450938	chr1	30231625	30231707	82	83	0	0	86.59
chr18	64219658	64219739	chr17	61180664	61180745	82	82	0	0	92.68
chr2	115513764	115513845	chr13	1526007	1526088	82	82	0	1	93.9
chr2	126864874	126864955	chr13	12958861	12958942	82	82	0	0	100
chr6	9854774	9854855	chr5	10069236	10069317	82	82	0	0	97.56
chr7	66347404	66347485	chr6	68152402	68152483	82	82	0	81	96.34
chr9	134879177	134879258	chr11	119762623	119762704	82	82	0	0	86.59
chr19	15740196	15740277	chr20	16365422	16365502	82	81	0	0	86.42
chr9	71697946	71698026	chr11	55521045	55521130	81	86	0	0	98.77
chr11	61362468	61362548	chr9	62336942	62337022	81	81	0	0	88.89
chr13	25958210	25958290	chr14	25133241	25133321	81	81	0	0	92.59
chr3	13030609	13030689	chr2	13485456	13485536	81	81	0	0	96.3
chr4	108792132	108792212	chr3	125277174	125277254	81	81	0	0	83.95
chrX	127666611	127666691	chrX	131887721	131887801	81	81	0	0	93.83
chr19	5599163	5599243	chr20	5889121	5889199	81	79	0	0	84.81
chr2	38546775	38546854	chr12	40021720	40022108	80	389	0	0	87.18
chr2	140015318	140015397	chr13	29041393	29041472	80	80	0	1	96.25

chr7	138057463	138057542	chr6	140819032	140819111	80	80	0	0	91.25
chr20	58799112	58799190	chr21	61799521	61799601	79	81	0	0	89.87
chr13	43030632	43030710	chr14	42741732	42741811	79	80	0	0	85.71
chr3	171970889	171970967	chr2	175465211	175465290	79	80	0	0	88.61
chr7	115305764	115305842	chr6	117583458	117583537	79	80	0	0	89.74
chr1	41681207	41681285	chr1	42642758	42642836	79	79	0	0	74.68
chr1	75982134	75982212	chr1	74542773	74542851	79	79	0	7	92.41
chr13	66222497	66222575	chr14	48618702	48618780	79	79	0	0	96.2
chr3	6009403	6009481	chr2	6225406	6225484	79	79	0	0	87.34
chr6	126137903	126137981	chr5	128654225	128654303	79	79	0	0	100
chr7	114585777	114585855	chr6	116666470	116666548	79	79	0	0	96.2
chrX	120970353	120970431	chrX	125060966	125061044	79	79	0	0	96.2
chr19	57460015	57460092	chr20	54865563	54865981	78	419	0	0	79.49
chr11	6692464	6692541	chr9	6872812	6872891	78	80	0	0	88.46
chrX	17576233	17576310	chrX	18170039	18170116	78	78	0	0	89.74
chr3	12698978	12699055	chr2	13153577	13153653	78	77	0	10	94.67
chr4	41705915	41705992	chr3	44392757	44392833	78	77	0	81	90.91
chr19	39931547	39931624	chr20	36279033	36279097	78	65	0	0	90.77
chr1	20836415	20836492	chr1	21158235	21158290	78	56	0	0	91.07
chr20	58799191	58799267	chr21	61799520	61799600	77	81	0	0	89.61
chr13	30597986	30598062	chr14	29804972	29805048	77	77	0	0	94.81
chr19	15754280	15754356	chr20	16350130	16350206	77	77	0	0	92.21
chr22	45302921	45302997	chr23	45624523	45624599	77	77	0	0	98.7
chr4	69973706	69973782	chr3	76912821	76912897	77	77	0	72	98.7
chrX	101980077	101980153	chrX	105492286	105492361	77	76	0	0	77.63
chr21	45192831	45192906	chr22	45367418	45367494	76	77	0	0	92.11
chr13	99495213	99495288	chr14	82824593	82824668	76	76	0	0	89.47
chr2	198139356	198139431	chr13	88196475	88196550	76	76	0	0	96.05
chr4	43625598	43625673	chr3	46321694	46321769	76	76	0	0	93.42
chr4	115975470	115975545	chr3	132653277	132653352	76	76	0	0	97.37
chr5	124980636	124980711	chr4	131299674	131299749	76	76	0	2	96.05
chr8	56267249	56267324	chr7	57960848	57960923	76	76	0	0	100
chr9	130860404	130860479	chr11	115738461	115738536	76	76	0	0	100
chrX	36029055	36029130	chrX	37387628	37387703	76	76	0	0	93.42
chr19	48565008	48565082	chr20	45469432	45469507	75	76	10	10	96
chr1	214403471	214403545	chr1	198139012	198139086	75	75	0	0	92
chr11	59044170	59044244	chr9	59943991	59944065	75	75	0	4	97.33
chr7	93869469	93869543	chr6	95471090	95471164	75	75	0	0	93.33
chr7	145852249	145852323	chr6	148692891	148692965	75	75	0	2	92
chr6	62835328	62835402	chr5	63542800	63542872	75	73	0	0	94.52
chr12	130530820	130530894	chr10	133698826	133698896	75	71	0	4	95.77
chr2	117287372	117287446	chr13	3344043	3344113	75	71	0	10	90.14
chr2	47311482	47311555	chr12	49345033	49345108	74	76	0	0	93.24
chr11	64145575	64145648	chr9	65363216	65363289	74	74	0	0	95.95
chr11	123685738	123685811	chr9	125711479	125711552	74	74	0	0	95.95
chr4	98430472	98430545	chr3	114760261	114760334	74	74	0	0	98.65
chr16	73499157	73499229	chr18	67825152	67825549	73	398	0	76	90.14
chr20	45551054	45551126	chr21	48098763	48098836	73	74	0	79	91.78
chr4	115813691	115813763	chr3	132491598	132491671	73	74	0	0	86.3
chr1	155202828	155202900	chr1	137291160	137291232	73	73	0	0	83.56
chr1	236078689	236078761	chr1	220050481	220050553	73	73	0	0	91.78
chr13	69361686	69361758	chr14	51793638	51793710	73	73	0	0	94.52
chrX	21569211	21569283	chrX	22329553	22329625	73	73	0	0	94.52

chr6	79194809	79194881	chr5	80679324	80679395	73	72	0	0	91.67
chr1	33520374	33520445	chr1	34255992	34256063	72	72	0	0	95.83
chr1	65675422	65675493	chr1	64014742	64014813	72	72	0	40	90.28
chr1	211771621	211771692	chr1	195484160	195484231	72	72	0	0	100
chr11	39573385	39573456	chr9	40043116	40043187	72	72	0	0	100
chr14	43556494	43556565	chr15	42760272	42760343	72	72	0	0	94.44
chr17	60917571	60917642	chr19	64174055	64174126	72	72	0	0	90.28
chr20	17404058	17404129	chr21	17253555	17253626	72	72	0	0	90.28
chr3	186554891	186554962	chr2	190561327	190561398	72	72	0	0	95.83
chr4	104086808	104086879	chr3	120489010	120489081	72	72	0	0	100
chr7	92912551	92912622	chr6	94494105	94494173	72	69	0	0	95.65
chr2	36865952	36866022	chr12	38321000	38321072	71	73	0	0	91.55
chr17	78590276	78590346	chr19	82401414	82401484	71	71	0	0	91.55
chr18	60799148	60799218	chr17	57660612	57660682	71	71	0	0	90.14
chrX	124079820	124079890	chrX	128206001	128206070	71	70	0	2	94.29
chr1	223026799	223026868	chr1	206846493	206846562	70	70	0	1	100
chr20	55731093	55731162	chr21	58660684	58660753	70	70	0	0	98.57
chr11	81889188	81889256	chr9	83270760	83270828	69	69	0	0	95.65
chr15	60147850	60147918	chr16	60682332	60682400	69	69	0	0	95.65
chr9	120884452	120884520	chr11	105623327	105623395	69	69	0	0	98.55
chrX	130914303	130914371	chrX	135336183	135336236	69	54	0	0	83.33
chr14	84326376	84326444	chr15	84452444	84452489	69	46	0	77	93.18
chr18	56007044	56007111	chr17	52887883	52887953	68	71	0	0	91.18
chr2	208168752	208168819	chr13	98215969	98216036	68	68	0	0	98.53
chr6	28193108	28193175	chr5	28649103	28649170	68	68	0	74	94.12
chr8	67240770	67240837	chr7	69247946	69248013	68	68	0	0	91.18
chr1	101529434	101529500	chr1	100578437	100578503	67	67	0	0	98.51
chr2	11190702	11190768	chr12	11910740	11910806	67	67	0	0	89.55
chr2	41572394	41572460	chr12	43138351	43138417	67	67	0	0	98.51
chr4	176196416	176196482	chr3	194175083	194175149	67	67	0	51	85.07
chr7	29414352	29414418	chr6	30565276	30565342	67	67	0	0	92.54
chr7	75478985	75479051	chr6	76578106	76578172	67	67	0	0	95.52
chr8	85564579	85564645	chr7	87701993	87702059	67	67	0	0	86.57
chr8	114444449	114444515	chr7	116966718	116966784	67	67	0	0	98.51
chr9	12113082	12113148	chr11	12308323	12308389	67	67	0	0	98.51
chr12	43824241	43824307	chr10	44439993	44440058	67	66	0	0	93.75
chr20	53312152	53312218	chr21	56172476	56172535	67	60	0	0	98.33
chr2	228305142	228305207	chr13	118904374	118904454	66	81	0	0	93.85
chr1	163716003	163716068	chr1	145955090	145955155	66	66	0	0	89.39
chr6	28381042	28381107	chr5	28849749	28849814	66	66	0	0	100
chr7	93450729	93450794	chr6	95047744	95047809	66	66	0	0	98.48
chr11	115507899	115507963	chr9	117504179	117504243	65	65	0	0	92.31
chr12	73025931	73025995	chr10	74751119	74751183	65	65	0	0	95.38
chr17	19426865	19426929	chr19	19762093	19762157	65	65	0	0	96.92
chr4	259899	259963	chr3	248928	248992	65	65	0	0	96.92
chr11	131430121	131430184	chr9	133580180	133580243	64	64	0	0	95.31
chr14	85009024	85009087	chr15	85167974	85168037	64	64	0	0	100
chr15	87841670	87841733	chr16	88714979	88715042	64	64	0	0	98.44
chrX	98523052	98523115	chrX	101766559	101766622	64	64	0	0	96.88
chr1	193671246	193671309	chr1	176313829	176313891	64	63	0	0	98.41
chr1	3299125	3299187	chr1	3151333	3151396	63	64	0	0	100
chr1	154700655	154700717	chr1	136712341	136712403	63	63	0	0	95.24
chr18	50757719	50757781	chr17	47384412	47384474	63	63	0	41	84.13

chr4	8661058	8661120	chr3	8739127	8739189	63	63	0	0	96.83
chr4	172344414	172344476	chr3	190189192	190189254	63	63	0	0	98.41
chr8	3933658	3933720	chr7	4107814	4107876	63	63	0	0	92.06
chr18	23616631	23616692	chr17	19550425	19550487	62	63	0	0	90.32
chr1	81018908	81018969	chr1	79629963	79630024	62	62	0	0	96.77
chr18	44649627	44649688	chr17	41153902	41153963	62	62	0	0	88.71
chr5	137088186	137088247	chr4	143688181	143688242	62	62	0	0	95.16
chr1	99798344	99798404	chr1	98846324	98846384	61	61	0	0	91.8
chr13	67393137	67393197	chr14	49800203	49800263	61	61	0	0	93.44
chr22	34517535	34517595	chr23	34644345	34644405	61	61	0	0	100
chr3	9084573	9084633	chr2	9453601	9453661	61	61	0	0	86.89
chr3	46118052	46118112	chr2	47468855	47468915	61	61	0	0	96.72
chr3	129357522	129357582	chr2	131249275	131249335	61	61	0	0	98.36
chr6	30252358	30252418	chr5	30631208	30631268	61	61	0	1	98.36
chr5	161224319	161224379	chr4	168405616	168405675	61	60	0	11	93.33
chr1	209091889	209091948	chr1	192731199	192731258	60	60	0	25	88.33
chr11	1608026	1608085	chr9	1742595	1742654	60	60	0	0	95
chr12	128374582	128374641	chr10	131504736	131504795	60	60	0	0	85
chr18	47349635	47349694	chr17	43943742	43943801	60	60	0	0	89.47
chr2	67073405	67073464	chr12	69315798	69315857	60	60	0	0	98.33
chr2	143223140	143223199	chr13	32258754	32258813	60	60	0	0	100
chr4	177242033	177242092	chr3	195205651	195205710	60	60	0	0	88.33
chr6	109447381	109447440	chr5	111756127	111756186	60	60	0	0	90
chr9	106118694	106118753	chr11	90572383	90572442	60	60	0	0	91.67
chrX	17310519	17310578	chrX	17894024	17894083	60	60	0	0	100
chr10	627318	627377	chr8	685061	685118	60	58	0	77	81.03
chr4	121222332	121222391	chr3	137911722	137911773	60	52	0	35	76.92
chr12	54815524	54815582	chr10	56063214	56063272	59	59	0	0	94.92
chr14	65067379	65067437	chr15	64673654	64673712	59	59	0	0	96.61
chr2	145463788	145463846	chr13	34499319	34499377	59	59	0	0	93.22
chr6	18823744	18823802	chr5	19196940	19196998	59	59	0	0	98.31
chr8	35131019	35131077	chr7	36332161	36332219	59	59	0	0	100
chrX	41578014	41578072	chrX	42923271	42923329	59	59	0	0	86.44
chrX	133890969	133891027	chrX	138527804	138527862	59	59	0	3	86.44
chrX	98522851	98522909	chrX	101770764	101770813	59	50	0	0	92
chr7	62877729	62877786	chr6	64496295	64496355	58	61	0	0	79.31
chrX	112017440	112017497	chrX	115680659	115680719	58	61	0	0	87.93
chr20	61945584	61945641	chr21	65009860	65009918	58	59	0	0	86.21
chr14	85638372	85638429	chr15	85808713	85808770	58	58	0	0	96.55
chr19	58470173	58470230	chr20	55924032	55924089	58	58	0	0	82.76
chr2	13744538	13744595	chr12	14512866	14512923	58	58	0	0	100
chr2	182108367	182108424	chr13	71860394	71860451	58	58	0	0	89.66
chr9	71479785	71479842	chr11	55297566	55297623	58	58	0	57	82.76
chrX	3302803	3302860	chrX	3066965	3067022	58	58	0	71	86.21
chr10	124665156	124665213	chr8	126903556	126903607	58	52	0	0	86.27
chr10	44262415	44262471	chr8	44986556	44986615	57	60	0	0	100
chr10	34729793	34729849	chr8	35155381	35155437	57	57	0	0	96.49
chr14	28696851	28696907	chr15	27647637	27647693	57	57	0	0	94.74
chr19	39875758	39875814	chr20	36445665	36445721	57	57	0	0	94.74
chr6	170055777	170055833	chr5	174543050	174543106	57	57	0	0	85.96
chr2	99187855	99187911	chr12	101995279	101995334	57	56	0	0	100
chr18	63800683	63800738	chr17	60746817	60746872	56	56	0	0	98.21
chr2	134276263	134276318	chr13	23279992	23280047	56	56	0	0	91.07

chr3	42897343	42897398	chr2	44099429	44099484	56	56	0	0	87.5
chrX	92681323	92681378	chrX	95576635	95576690	56	56	0	49	92.86
chr1	234872015	234872069	chr1	218760208	218760266	55	59	0	0	96.36
chr9	36245057	36245111	chr11	36857781	36857836	55	56	0	0	98.18
chr13	69198486	69198540	chr14	51617592	51617646	55	55	0	0	96.36
chr13	86767394	86767448	chr14	69511965	69512019	55	55	0	0	98.18
chr4	7729208	7729262	chr3	7804002	7804056	55	55	0	0	100
chr7	73391752	73391806	chr6	75138051	75138105	55	55	0	0	87.27
chr5	670715	670768	chr4	777453	777507	54	55	0	0	96.3
chr2	64721050	64721103	chr12	66953510	66953563	54	54	0	0	100
chr20	9027150	9027203	chr21	8917109	8917162	54	54	0	0	90.74
chr21	44929053	44929106	chr22	45076248	45076301	54	54	0	0	96.3
chr4	13685569	13685622	chr3	13917427	13917480	54	54	0	0	83.33
chr4	108792078	108792131	chr3	125277174	125277227	54	54	0	0	85.19
chr4	188325852	188325905	chr3	206538782	206538835	54	54	0	0	96.3
chr5	600112	600165	chr4	707258	707311	54	54	0	0	100
chr7	154413197	154413250	chr6	157256061	157256114	54	54	0	0	92.59
chrX	68270243	68270296	chrX	70307900	70307953	54	54	0	0	88.89
chr1	150223787	150223839	chr1	132180518	132180570	53	53	0	0	69.81
chr1	183502497	183502549	chr1	166086183	166086235	53	53	0	0	94
chr21	45473858	45473910	chr22	45655531	45655583	53	53	0	0	100
chr4	69999340	69999392	chr3	76887328	76887380	53	53	0	0	100
chr6	165915178	165915230	chr5	170230184	170230236	53	53	0	0	98.11
chr16	6846032	6846083	chr18	6963805	6963856	52	52	0	0	90.38
chrX	99268013	99268064	chrX	102513960	102514011	52	52	0	0	92.31
chr3	112768688	112768739	chr2	114356782	114356830	52	49	0	5	91.84
chr1	185323746	185323796	chr1	167898120	167898170	51	51	0	0	100
chr10	11939804	11939854	chr8	12079784	12079834	51	51	0	0	98.04
chr3	155280656	155280706	chr2	158409840	158409890	51	51	0	0	96.08
chr6	154033174	154033224	chr5	157559629	157559679	51	51	0	0	96.08
chr7	87257858	87257908	chr6	88774607	88774657	51	51	0	0	98.04
chr9	125355797	125355847	chr11	110158028	110158078	51	51	0	0	92.16
chr9	127025568	127025618	chr11	111836220	111836270	51	51	0	0	94.12
chr6	51978613	51978663	chr5	53737377	53737426	51	50	0	0	98
chr11	109215304	109215353	chr9	111172648	111172697	50	50	0	0	98
chr17	4332901	4332950	chr19	4641603	4641652	50	50	0	0	90
chr4	12759227	12759276	chr3	12969712	12969761	50	50	0	38	98
chr4	69978997	69979046	chr3	76907557	76907606	50	50	0	0	100
chr9	126312707	126312756	chr11	111119352	111119401	50	50	0	10	86
chrX	81134242	81134291	chrX	83676980	83677029	50	50	0	0	96
chrX	70743479	70743528	chrX	73010848	73010895	50	48	0	89	95.83
chr19	56684189	56684237	chr20	54111127	54111175	49	49	0	0	100
chr21	44993921	44993969	chr22	45172449	45172497	49	49	0	0	83.67
chr3	45884442	45884490	chr2	47231954	47232002	49	49	0	0	97.96
chr3	52200544	52200592	chr2	53813474	53813522	49	49	0	0	91.84
chr8	29961251	29961299	chr7	31355140	31355188	49	49	0	0	91.84
chr1	243618194	243618241	chr1	227742369	227742416	48	48	0	0	93.75
chr2	65508441	65508488	chr12	67748286	67748333	48	48	0	0	100
chr20	48154620	48154667	chr21	50773544	50773591	48	48	0	2	87.5
chr21	42468811	42468858	chr22	42590037	42590084	48	48	0	0	97.92
chr1	55023793	55023840	chr1	53308717	53308762	48	46	0	0	86.96
chr13	106212898	106212944	chr14	89617274	89617320	47	47	0	0	97.87
chr16	83939177	83939223	chr18	78861948	78861994	47	47	0	0	93.62

chr2	22690471	22690517	chr12	23568702	23568748	47	47	0	0	93.62
chr15	43029150	43029195	chr16	42955493	42955538	46	46	0	60	97.83
chr4	132232041	132232086	chr3	149202936	149202981	46	46	0	0	86.96
chr9	110243521	110243564	chr11	94770731	94770775	44	45	0	0	95.12
chr1	222318412	222318455	chr1	206133189	206133232	44	44	0	0	79.55
chr18	42034867	42034910	chr17	38372288	38372331	44	44	0	0	95.45
chr9	15711610	15711653	chr11	15950048	15950091	44	44	0	0	100
chr13	93653393	93653436	chr14	76681152	76681193	44	42	0	0	100
chr10	52400939	52400981	chr8	53280558	53280600	43	43	0	0	95.35
chr11	82645498	82645540	chr9	84063837	84063879	43	43	0	26	93.02
chr16	17773600	17773642	chr18	18152851	18152893	43	43	0	80	86.05
chr16	73978422	73978464	chr18	68376071	68376113	43	43	0	0	90.7
chr5	1199482	1199524	chr4	1240525	1240567	43	43	0	0	86.05
chr5	178296692	178296734	chr4	185805610	185805652	43	43	0	0	93.02
chrX	118887526	118887568	chrX	122959331	122959373	43	43	0	19	90.7
chr8	115520605	115520647	chr7	118086697	118086736	43	40	0	0	82.5
chr16	31519351	31519392	chr18	32563777	32563822	42	46	0	0	95.24
chr18	43567831	43567872	chr17	39935737	39935779	42	43	0	0	95.24
chr10	55905670	55905711	chr8	56829472	56829513	42	42	0	0	100
chr11	55777214	55777255	chr9	56610633	56610674	42	42	0	0	85.71
chr15	49488502	49488543	chr16	49518568	49518609	42	42	0	0	100
chr18	23414717	23414758	chr17	19347177	19347218	42	42	0	0	95.24
chr2	108336113	108336154	chr12	116087478	116087519	42	42	0	0	100
chr22	46121653	46121694	chr23	46461818	46461859	42	42	0	0	83.33
chr3	14616294	14616335	chr2	15214780	15214821	42	42	0	0	95.24
chr1	218037054	218037095	chr1	201783733	201783772	42	40	0	0	100
chr18	19517242	19517282	chr17	15420020	15420063	41	44	0	0	92.68
chr10	127121600	127121640	chr8	129640287	129640327	41	41	0	0	87.8
chr11	1608327	1608367	chr9	1742286	1742326	41	41	0	0	97.56
chr12	2659823	2659863	chr10	2733814	2733854	41	41	0	0	97.56
chr15	79464966	79465006	chr16	80131172	80131212	41	41	0	0	92.68
chr3	51823259	51823299	chr2	53394779	53394819	41	41	0	0	95.12
chr4	65134679	65134719	chr3	81507154	81507194	41	41	0	0	95.12
chr4	69978953	69978993	chr3	76907610	76907650	41	41	0	0	97.56
chr4	70142789	70142829	chr3	76743611	76743651	41	41	0	0	100
chr7	110956974	110957014	chr6	112902596	112902636	41	41	0	0	95.12
chrX	35624285	35624325	chrX	36918744	36918784	41	41	0	0	82.93
chr1	200332386	200332425	chr1	183263966	183264005	40	40	0	0	100
chr14	101992996	101993035	chr15	102599762	102599801	40	40	0	0	87.5
chr2	145623669	145623708	chr13	34666496	34666535	40	40	0	0	85
chr20	58799072	58799111	chr21	61799520	61799559	40	40	0	0	92.5
chr21	39863474	39863513	chr22	39906631	39906670	40	40	0	10	85
chr4	138735757	138735796	chr3	155913815	155913854	40	40	0	0	100
chr4	166315998	166316037	chr3	183669012	183669051	40	40	0	0	82.5
chr1	5721084	5721122	chr1	5600089	5600127	39	39	0	0	92.31
chr14	68184893	68184931	chr15	67839877	67839915	39	39	0	0	92.31

chr2	208168820	208168858	chr13	98218727	98218765	39	39	0	0	92.31
chr20	22483634	22483672	chr21	22429981	22430019	39	39	0	0	97.44
chr3	145968017	145968055	chr2	148669227	148669265	39	39	0	0	94.74
chr3	84393833	84393870	chr2	86672660	86672698	38	39	0	0	97.37
chr6	34122592	34122629	chr5	34570304	34570341	38	38	0	0	92.11
chr6	70815513	70815550	chr5	71950260	71950297	38	38	0	0	89.47
chr10	123400581	123400617	chr8	125724973	125725009	37	37	0	0	86.49
chr20	32771138	32771174	chr21	34777498	34777534	37	37	0	0	100
chr7	112030119	112030155	chr6	114045610	114045646	37	37	0	0	100
chr11	2485811	2485846	chr9	2669346	2669381	36	36	0	0	100
chr21	45473817	45473852	chr22	45655589	45655624	36	36	0	0	97.22
chrX	111092795	111092830	chrX	114829964	114829999	36	36	0	0	100
chr5	4994052	4994086	chr4	5101968	5102002	35	35	0	0	91.43
chr4	4589540	4589573	chr3	4597804	4597837	34	34	0	0	85.29
chr6	126174671	126174704	chr5	128693465	128693498	34	34	0	0	94.12
chr9	71764733	71764766	chr11	55586131	55586164	34	34	0	0	100
chrX	54699580	54699613	chrX	55991202	55991235	34	34	0	0	94.12
chr1	21806481	21806514	chr1	22148076	22148107	34	32	0	0	96.88
chr1	220551575	220551607	chr1	204359251	204359283	33	33	0	0	96.97
chr6	114090698	114090730	chr5	116415376	116415408	33	33	0	0	96.97
chrX	56220198	56220230	chrX	57642039	57642071	33	33	0	0	93.94
chrX	152412735	152412767	chrX	157473694	157473726	33	33	0	0	96.97
chr12	55018042	55018073	chr10	56269738	56269769	32	32	0	0	96.88
chr13	100734798	100734828	chr14	84071654	84071684	31	31	0	0	96.77
chr2	62105635	62105665	chr12	64322732	64322762	31	31	0	0	100
chr9	107319504	107319534	chr11	91785749	91785779	31	31	0	0	93.55
chr11	1562812	1562841	chr9	1750987	1751016	30	30	0	0	96.66
chr3	38647712	38647741	chr2	39750404	39750433	30	30	0	0	100
chr4	136671147	136671176	chr3	153716686	153716715	30	30	0	0	100
chr7	143223639	143223668	chr6	145973634	145973663	30	30	0	0	96.67
chr10	71123689	71123717	chr8	72283257	72283286	29	30	0	0	96.55
chr13	113932615	113932643	chr14	97584250	97584278	29	29	0	0	82.76
chr4	34391479	34391507	chr3	34794727	34794755	29	29	0	0	89.66
chr5	159864771	159864799	chr4	167044302	167044330	29	29	0	89	93.1
chr6	39374545	39374572	chr5	39938099	39938126	28	28	0	0	100
chr9	87934243	87934270	chr11	71931102	71931129	28	28	0	0	92.86
chr7	39356827	39356853	chr6	45163135	45163163	27	29	0	0	100
chr10	36670667	36670693	chr8	37197747	37197773	27	27	0	0	92.59
chr6	133940110	133940136	chr5	136581153	136581179	27	27	0	0	92.59
chr2	135518286	135518311	chr13	24508333	24508358	26	26	0	0	100
chr9	8188514	8188539	chr11	8114781	8114806	26	26	0	0	100
chr1	215324644	215324667	chr1	199073762	199073785	24	24	0	0	95.83
chr10	19802073	19802096	chr8	19945482	19945505	24	24	0	0	95.83
chr2	108336369	108336392	chr12	116087240	116087263	24	24	0	0	100
chr3	30797191	30797214	chr2	31779002	31779025	24	24	0	0	100
chr9	8188547	8188570	chr11	8114750	8114773	24	24	0	0	100
chr19	38699394	38699416	chr20	35215146	35215168	23	23	0	0	91.3

Table S1
A list of all 1576 variants that were identified between the chimpanzee and human assemblies, after applying filtering criteria
The column "% Dup Coverage" shows the fraction of each inversion region that is covered by segmental duplications.
The column "% Repeat Coverage" shows the fraction of each inversion region that is covered by repeats as defined by RepeatMasker.
Some larger inversions are fragmented in the chimpanzee assembly. See Table S2A for a curated list of inversions >25kb.
